# Comprehensive sequencing profile and functional analysis of IsomiRs in human pancreatic islets and beta cells

**DOI:** 10.1007/s00125-025-06397-4

**Published:** 2025-03-18

**Authors:** Stefano Auddino, Elena Aiello, Giuseppina E. Grieco, Daniela Fignani, Giada Licata, Marco Bruttini, Alessia Mori, Andrea F. Berteramo, Erika Pedace, Laura Nigi, Caterina Formichi, Claudiane Guay, Giuseppe Quero, Vincenzo Tondolo, Gianfranco Di Giuseppe, Laura Soldovieri, Gea Ciccarelli, Andrea Mari, Andrea Giaccari, Teresa Mezza, Agnese Po, Romano Regazzi, Francesco Dotta, Guido Sebastiani

**Affiliations:** 1 Diabetes Unit, Department of Medicine, Surgery and Neurosciences, University of Siena, Siena, Italy; 2 Fondazione Umberto Di Mario ONLUS c/o Toscana Life Science, Siena, Italy; 3 Tuscany Centre for Precision Medicine (CReMeP), Siena, Italy; 4 Department of Fundamental Neurosciences, University of Lausanne, Lausanne, Switzerland; 5 Dipartimento di Medicina e Chirurgia Traslazionale, Università Cattolica del Sacro Cuore, Rome, Italy; 6 Chirurgia Digestiva, Fondazione Policlinico Universitario Agostino Gemelli IRCCS, Rome, Italy; 7 General Surgery Unit, Fatebenefratelli Isola Tiberina-Gemelli Isola, Rome, Italy; 8 Endocrinologia e Diabetologia, Fondazione Policlinico Universitario Agostino Gemelli IRCCS, Rome, Italy; 9 Institute of Neuroscience, National Research Council, Padua, Italy; 10 Department of Molecular Medicine, Sapienza University of Rome, Rome, Italy; 11 Department of Biomedical Sciences, University of Lausanne, Lausanne, Switzerland

**Keywords:** Beta cell, Beta cell function, Human pancreatic islets, Insulin secretion, IsomiRs, MicroRNAs, Non-coding RNAs

## Abstract

**Aims/hypothesis:**

MiRNAs regulate gene expression, influencing beta cell function and pathways. Isoforms of miRNA (isomiRs), sequence variants of miRNAs with post-transcriptional modifications, exhibit cell-type-specific expression and functions. Despite their biological significance, a comprehensive isomiR profile in human pancreatic islets and beta cells remains unexplored. This study aims to profile isomiR expression in four beta cell sources: (1) laser capture microdissected human islets (LCM-HI); (2) collagenase-isolated human islets (CI-HI); (3) sorted beta cells; and (4) the EndoC-βH1 beta cell line, and to investigate their potential role in beta cell function.

**Methods:**

Small RNA-seq and/or small RNA dataset analysis was conducted on human pancreatic islets and beta cells. Data were processed using the sRNAbench bioinformatics pipeline to classify isomiRs based on sequence variations. A beta cell-specific isomiR signature was identified via cross-validation across datasets. Correlations between LCM-HI isomiR expression and in vivo clinical parameters were analysed using regression models. Functional validation of isomiR-411-5p-Ext5p(+1) was performed via overexpression in EndoC-βH1 cells and CI-HI, followed by glucose-stimulated insulin secretion (GSIS) assays and/or transcriptomic analysis.

**Results:**

IsomiRs constituted 59.2 ± 1.9% (LCM-HI), 59.6 ± 2.4% (CI-HI), 42.3 ± 7.2% (sorted beta cells) and 43.8 ± 1.2% (EndoC-βH1) of total miRNA reads (data represented as mean ± SD), with 3′ end trimming (Trim3p) being the predominant modification. A beta cell-specific isomiR signature of 30 sequences was identified, with isomiR-411-5p-Ext5p(+1) showing a significant inverse correlation with basal insulin secretion (*p*=0.0009, partial *R*^2^=0.68) and total insulin secretion (*p*=0.005, partial *R*^2^=0.54). Overexpression of isomiR-411-5p-Ext5p(+1), but not of its canonical counterpart, importantly reduced GSIS by 51% ( ± 15.2%; mean ± SD) (*p*=0.01) in EndoC-βH1 cells. Transcriptomic analysis performed in EndoC-βH1 cells and CI-HI identified 47 genes significantly downregulated by isomiR-411-5p-Ext5p(+1) (false discovery rate [FDR]<0.05) but not by the canonical miRNA, with enriched pathways related to Golgi vesicle biogenesis (FDR=0.017) and trans-Golgi vesicle budding (FDR=0.018). TargetScan analysis confirmed seed sequence-dependent target specificity for 81 genes uniquely regulated by the isomiR (*p*=1.1 × 10⁻⁹).

**Conclusions/interpretation:**

This study provides the first comprehensive isomiR profiling in human islets and beta cells, revealing their substantial contribution to miRNA regulation. IsomiR-411-5p-Ext5p(+1) emerges as a distinct key modulator of insulin secretion and granule dynamics in beta cells. These findings highlight isomiRs as potential biomarkers and therapeutic targets for diabetes, warranting further exploration of their roles in beta cell biology.

**Graphical Abstract:**

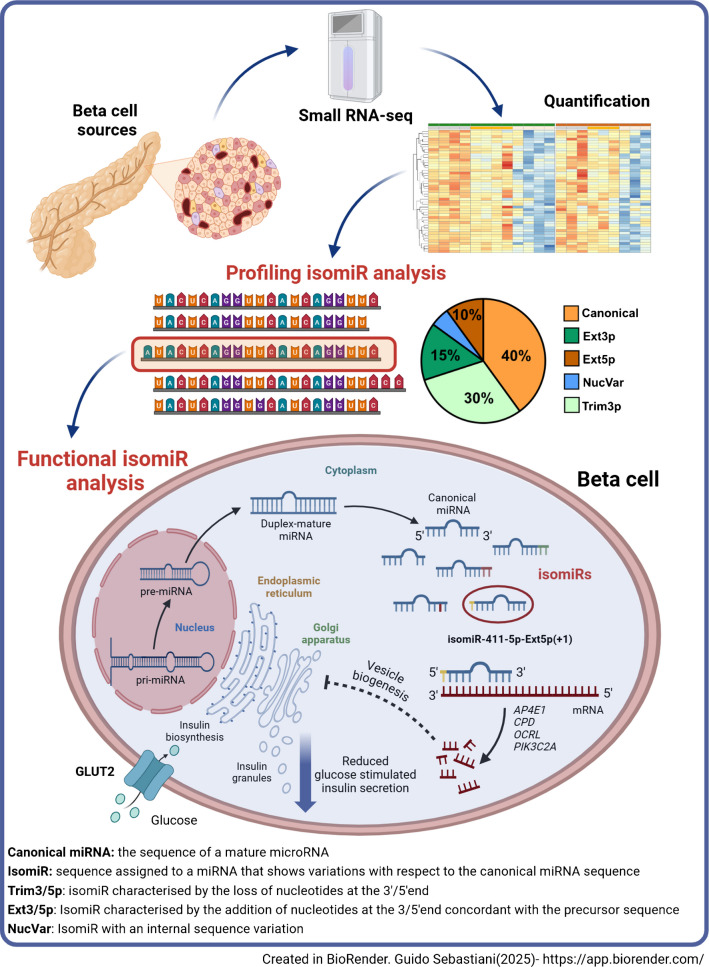

**Supplementary Information:**

The online version of this article (10.1007/s00125-025-06397-4) contains peer-reviewed but unedited supplementary material.


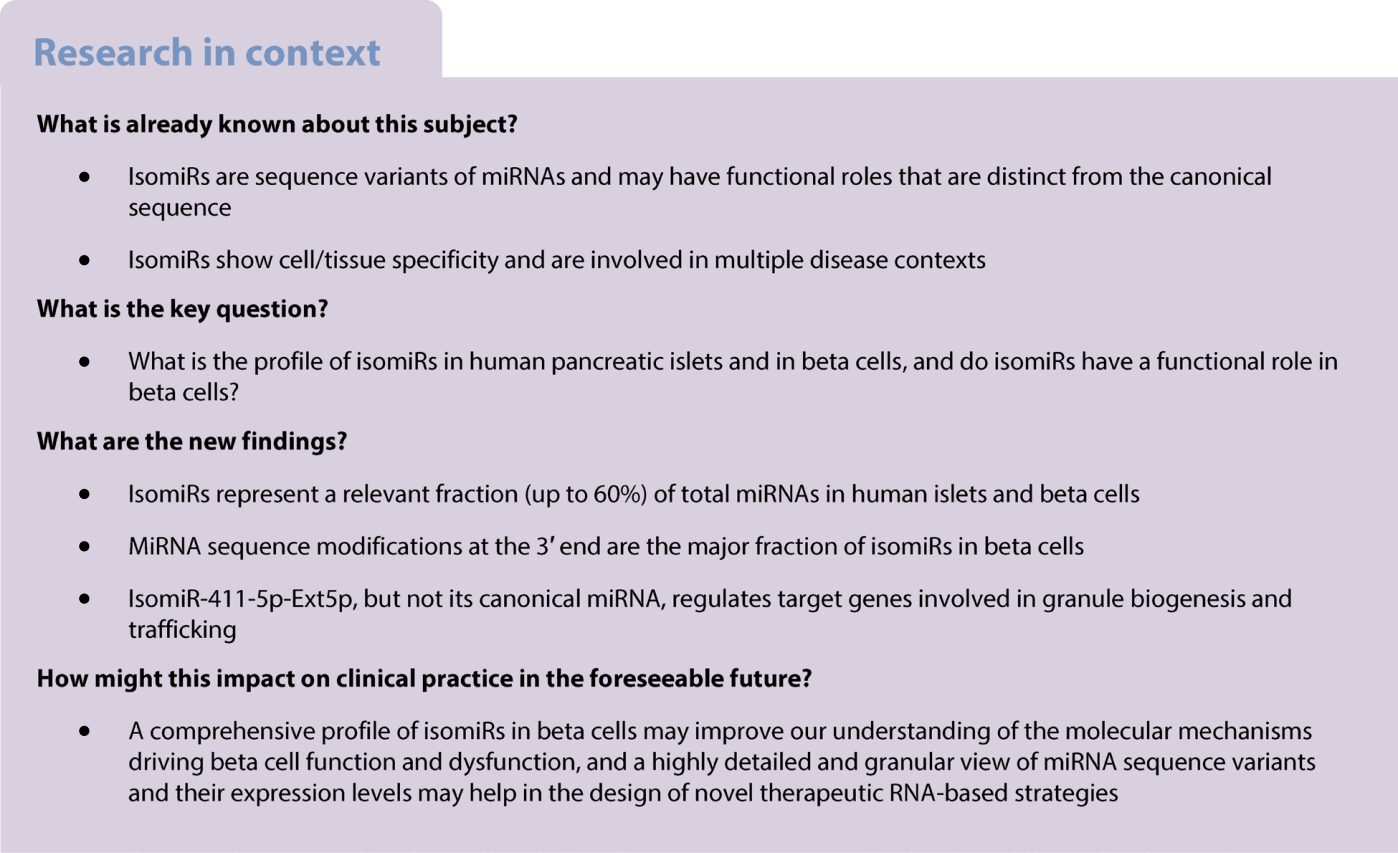


## Introduction

MiRNAs are ~22 nucleotide-long non-coding RNA transcripts involved in the regulation of gene expression by pairing to mRNA target sequences [1–3]. The major determinant of miRNAs binding to target mRNAs is a 6–8 nucleotide-long domain at the 5′ end of miRNA (the seed sequence). The exact pairing between the miRNA seed sequence and the corresponding mRNA target sequence most likely results in the reduction of target mRNA expression. Hence, the targetome of each miRNA is largely determined by seed sequence. Consequently, in light of their major role in the modulation of gene expression, it is likely that miRNAs regulate pivotal biological processes [2] and, as many of them are altered in multiple diseases, they should be considered as potential therapeutic targets [4].

Known and mapped miRNAs are listed in public databases, such as miRbase (www.miRbase.org) [5]. MiRNAs included in miRbase have been defined as a single sequence of RNA (i.e. ‘canonical’ miRNA). However, next-generation sequencing (NGS) studies performed in multiple biological contexts have revealed a relevant proportion of miRNA sequences that differed in length and/or one or more nucleotides from canonical miRNAs [6–8]. MiRNA sequence variants, called isoforms of miRNA (isomiRs), were initially dismissed as sequencing errors or artefacts. Nevertheless, the refinement of sequencing technologies and bioinformatic analytical pipelines have confirmed that these are *bona fide* miRNA sequence variants [9, 10].

The biogenesis of isomiRs is strictly related to the maturation and/or editing of precursor or mature canonical miRNAs and involves alternative miRNA precursor (pre-miRNA) cropping and/or dicing, terminal nucleotide trimming or addition (5′ or 3′ trimming/extension), non-templated nucleotide addition (NTA) and single nucleotide modification due to RNA editing, all operated by specialised enzymes. However, the exact molecular mechanism of their biogenesis remains unclear. Nonetheless, isomiRs have been shown to be tissue specific [11] and, for example, can be used to classify multiple cancer types [12]. Of importance, isomiRs can be functionally associated with the RNA-induced silencing complex (RISC), and thus able to inhibit mRNA expression [13, 14]. The function of isomiRs may vary with respect to canonical miRNA depending on the sequence variation type, thus broadening the role of a specific miRNA or having a completely independent role in regulating physiological or pathological processes. Particularly, variations at the 5′ end of canonical miRNAs (both trimming and extensions) can modify the seed sequence thus changing its targetome; alternatively, trimming or extension at the 3′ end of canonical miRNAs can modify its stability/half-life, Argonaute 2 (AGO2) protein loading or non-canonical target recognition, thus significantly impacting on final function.

The advent of high-throughput analytical platforms has bolstered the characterisation of miRNAs expression profiles in multiple cells and tissues [15], revealing a landscape of ubiquitous and/or cell/tissue-specific miRNAs and isomiRs [16]. Multiple studies also described the profile of miRNAs in pancreatic islets and in beta cells, showing a peculiar fingerprint characterised by the high expression of enriched miRNAs (i.e. miR-375, miR-7) alongside others involved in the regulation of specialised endocrine islet cell functions [17–20]. Of note, additional studies have further clarified the role of these miRNAs in beta cell function, demonstrating their activity in development and differentiation [21–24], proliferation [25–29], apoptosis [30–36] and, importantly, basal- and/or glucose-stimulated insulin secretion (GSIS) [17, 37–44].

Given the growing diversity in both the sequence and functional roles of each miRNA, it is crucial to re-examine their expression patterns at the sequence level in human pancreatic islets and beta cells. This re-evaluation aims to deepen our understanding of the molecular mechanisms underlying miRNA regulation of beta cell functions and may shed light on novel mechanisms that can be therapeutically treated. By distinguishing canonical miRNA sequences from their variants, isomiRs introduce additional complexity to the field, offering new perspectives on the regulation of physiological processes, including beta cell function [45]. Although there have been previous indications of isomiRs playing a role in pancreatic islets [46], a comprehensive characterisation of isomiRs, especially considering recent advances in technological platforms and bioinformatic pipelines, has yet to be undertaken.

In this study, we analysed isomiRs in multiple small RNA-seq experiments or datasets, with the aim of establishing a distinct signature of isomiRs specific to pancreatic islets and beta cells. We employed an up-to-date NGS protocol and applied a rigorous bioinformatic pipeline to analyse a robust and reliable set of isomiRs.

## Methods

### Study donors and human pancreas tissue collection

Human pancreatic tissue sections were obtained from pancreases of *n*=19 donors, specifically from *n*=3 brain-dead adult non-diabetic multiorgan donors (INNODIA EUnPOD network) and from *n*=16 non-diabetic living donors undergoing pylorus-preserving pancreatoduodenectomy at Agostino Gemelli University Hospital, Rome, Italy. Indications for surgery were periampullary tumours, pancreatic intraductal papillary tumours, mucinous cystic neoplasm of the pancreas and nonfunctional pancreatic neuroendocrine tumours (electronic supplementary material [ESM] Table 1). Tissue specimens were snap-frozen and stored at −80°C. All donors provided informed consent, and the study protocol (ClinicalTrials.gov NCT02175459) was approved by the local ethics committees.

In the INNODIA EUnPOD network, pancreases not suitable for organ transplantation were obtained with informed written consent by organ donors’ next-of-kin and processed with the approval of the local ethics committee of the University of Pisa (ESM Table 2).

Living donors were metabolically profiled before undergoing surgery, as previously described [47, 48], and subjected to a 75 g OGTT and HbA_1c_ testing to exclude diabetes, according to the American Diabetes Association criteria. None of the participants enrolled had a family history of diabetes. For detailed methods regarding metabolic screening please refer to the ESM Methods section.

### OGTT

A standard 75 g OGTT was performed after a 12 h overnight fast with measurements of glucose, insulin and C-peptide at 0, 30, 60, 90 and 120 min after the glucose load. Based on the OGTT, we classified the population as normal glucose tolerant (NGT) if 2 h post-load glucose was below 7.8 mmol/l. During OGTT, insulin secretion was derived from C-peptide levels by deconvolution. Beta cell glucose sensitivity, i.e. the slope of the relationship between insulin secretion and glucose concentration, was estimated from the OGTT by modelling, as previously described [49, 50]. Rate sensitivity, also estimated from OGTT modelling, is a beta cell functional parameter that represents the dependence of the insulin secretion rate (ISR) on the rate of change in glucose concentration and is related to early insulin release.

### Laser capture microdissection of human pancreatic islets

Pancreatic human tissues were cryosectioned and subjected to laser capture microdissection (LCM) using an Arcturus XT Laser Capture Microdissection system (Arcturus Engineering, Mountain View, CA, USA) by melting thermoplastic films mounted on transparent LCM caps (cat. LCM0214, Thermo Fisher Scientific, Waltham, MA, USA) on specific islet areas. Intrinsic beta cell autofluorescence allowed the identification of human pancreatic islets for the LCM procedure (see ESM Methods).

### Human Islets and sorted beta cells

Human pancreatic islets (collagenase-isolated human islets [CI-HI]) used for small RNA-seq profiling or for transcriptomic analysis were obtained from non-diabetic brain-dead organ donors from Tebu-Bio (Le Perray-en-Yvelines, France) (ESM Table 3). After receipt, the islets were cultured in RPMI1640 (1X)+ GlutaMAX medium (5.5 mmol/l glucose) (cat.72400-021, Thermo Fisher Scientific) supplemented with 10% FBS (cat. ECS0180L, EuroClone, Milan, Italy) 1% antibiotic/antimycotic (cat. A5955, Sigma Aldrich, St Louis, MO, USA) and 1% l-glutamine (cat. G7513, Sigma Aldrich). After an overnight incubation at 28°C and 5% CO_2_, the islets were dissociated into single cells using trypsin-EDTA solution (cat. T3924, Sigma Aldrich) and plated at 1.5 × 10^5^ cells per well for transfection (see ESM Methods, Human Islets checklist). Alternatively, CI-HI were obtained from the Cell Isolation and Transplantation Center of the University of Geneva through the Breakthrough T1D (formerly JDRF) award 31-2008-416 (ECIT Islet for Basic Research program). CI-HI cells were cultured at 37°C and 5% CO_2_ for few days in CMRL 1066 medium supplemented with 10% FCS, 100 U/ml penicillin, and 100 mg/ml streptomycin, 2 mmol/l l-glutamine and 10 mmol/l HEPES (see ESM Methods, Human Islets checklist).

FASTQ files from sorted beta cell small RNA-seq were downloaded from the SRA database (SRR871671, SRR873401, SRR873410) (see ESM Methods). Beta cells from *n*=3 non-diabetic donors (ESM Table 4) were sorted by FACS based on high zinc content (see ESM Methods).

### EndoC-βH1 cell culture

EndoC-βH1 human beta cell line [51] was obtained from Human Cell Design (former UniverCell-Biosolutions) (Toulouse, France) and used between passages 74 and 88 for small RNA-seq and GSIS experiments and between passages 49 and 55 for RNA-seq. EndoC-βH1 cells were cultured at 37°C with 5%CO_2_ in a coated flask and maintained in culture in low-glucose DMEM (cat. D6046, Sigma Aldrich) (see ESM Methods). EndoC-βH1 cells were periodically tested for mycoplasma contamination using the MycoStrip mycoplasma detection kit (InvivoGen, San Diego, CA, USA).

### RNA isolation and quality control

RNA was isolated from LCM human islet (LCM-HI) samples using the PicoPure RNA isolation kit (cat. kit0204, Thermo Fisher Scientific), with DNase treatment for genomic DNA removal (cat. 79254, Qiagen, Hilden, Germany). RNA from human pancreatic islets and EndoC-βH1 cells was extracted using the Direct-zol RNA Microprep Kit (cat. R2062, Zymo Research, Irvine, CA, USA). RNA quality and integrity were assessed with the Agilent 2100 Bioanalyzer (cat. 5067-1513, Agilent Technologies, Santa Clara, CA, USA), and samples with an RNA integrity number (RIN) <5.0 were excluded (ESM Methods, ESM Table 5).

### Small RNA-seq

Small RNA-seq was performed using the QiaSeq miRNA library kit (cat. 331505, Qiagen), with Illumina NovaSeq, NextSeq or MiSeq platforms for sequencing. cDNA libraries were generated from 1 ng (from LCM-HI and EndoC-βH1 cell line) or 10 ng (CI-HI) of RNA and assessed for quality using Qubit spectrofluorometer (Qubit dsDNA HS Assay Kit, Thermo Fisher Scientific) and Bioanalyzer 2100 (Agilent High Sensitivity DNA Kit cat. 5067-4626). Sequencing libraries were pooled and denatured for sequencing with 75 × 1 single reads (see ESM Methods).

### Cell transfection

EndoC-βH1 cells and dispersed CI-HI were transfected with miRIDIAN miRNA mimics (miR-411-5p, isomiR-411-5p-Ext5p(+1), or scramble control) (Dharmacon, Lafayette, CO, USA) using Lipofectamine RNAiMAX (cat. 13778-150, Invitrogen, Waltham, MA, USA) (see ESM Methods).

### IsomiR quantification using ddPCR

To assess transfection, TaqMan advanced reverse transcription and miR-Amp (Life Technologies, CA, USA) was performed on EndoC-βH1 and CI-HI following the manufacturer’s instructions on 10 ng of extracted RNA. Then, miR-411-5p (TaqMan advanced assay cat. #A25576 ID478086_mir, Life Technologies) and isomiR-411-5p-Ext5p(+1) (custom assay 5′-ATAGTAGACCGTATAGCGTACG-3′) expression was evaluated through ddPCR detection on a BioRad QX200 system using a probes assay (BioRad, Mississauga, ON, Canada) (see ESM Methods).

### GSIS and ELISA assay

EndoC-βH1 cells were seeded at 3.75 × 10^5^ cells per well in 12 well plates, incubated at 37°C with 5% CO_2_, and transfected with scrambled control, miR-411-5p or isomiR-411-5p-Ext5p(+1) sequences (see ESM Methods). After transfection, cells were starved in ULTI-ST medium (Human Cell Design, Toulouse, France) overnight, followed by pre-incubation in KREBS (Human Cell Design) supplemented with BSA (cat. 10775835001, Sigma Aldrich) medium for 1 h. Cells were then stimulated with either 0 mmol/l (KREBS solution without glucose addition) or 20 mmol/l glucose for 40 min. The medium was collected, centrifuged, and stored at −20°C for up to 4 weeks before measuring secreted insulin. Intracellular insulin was assessed after cell lysis using a specific lysis buffer and stored similarly. Insulin levels were quantified using a human insulin ELISA Kit (Mercodia, Sweden) and calculated based on a standard curve (see ESM Methods).

### Bioinformatic pipeline for isomiR analysis and classification

FASTQ files obtained from the small RNA-seq experiments were analysed with the sRNAbench online pipeline [52] (https://arn.ugr.es/srnatoolbox/newbench/; accessed 20 Sep 2022 to 16 Jan 2024). Reads were processed for adapter trimming and PCR duplicate removal, with stringent quality control ensuring high base-level accuracy (Q ≥ 30). Reads were mapped to the human genome (GRCh38.p13) using Bowtie with specific parameters and annotated them using miRBase release 22.1 [5]. Low-quality and ambiguous sequences were filtered, followed by a relative abundance filter based on reads per million (RPM), with sequences contributing less than 1% removed (see ESM Methods). This filter, validated through an in silico simulation (see ESM Methods), was designed to eliminate sequencing errors. IsomiRs were classified into different classes according to the post-transcriptional modification compared with the canonical miRNA sequence: canonical, 3′ length variant (Lv3p), 5′ length variant (Lv5p), NTA, multi-length variant (MultiVariant) and nucleotide variant (NucVar). Both Lv3p and Lv5p were further classified as either trimmed at the 3ʹ or 5ʹ end (Trim3p/5p) or extended at the 3ʹ or 5ʹ end (Ext3p/5p). For each sequence, seed sequence conservation was assessed to distinguish isomiRs with different seed sequences, categorised as ‘not-conserved seed’ if differences were detected (see ESM Methods).

### Small RNA-seq data analysis

Filtered isomiR expression data were normalised with DESeq2 (Version 1.38.3) median of the ratios methods [53]. To assess the consistency of post-transcriptional modifications in beta cells, we focused on miRNAs with robust expression across all the *n*=4 beta cell and human islet sequencing experiments (mean expression >20 normalised counts) (LCM-HI, CI-HI, sorted beta cells and EndoC-βH1). For these miRNAs, we calculated the average proportion of each isomiR class and assigned each miRNA to the isomiR class contributing most to its total expression. This was visualised in a heatmap to evaluate isomiR class consistency across experiments (see ESM Methods). To assess isomiR specificity in beta cells, isomiR expression data from the isomiRdb repository (https://ccb-compute.cs.uni-saarland.de/isomirdb; accessed 28 Oct 2023) [54] were compared with data from the four beta cell and human islet sequencing experiments. The composition of each miRNA was computed and a principal components analysis (PCA) was then performed to assess the segregation of the beta cells compared with other cell types from isomiRdb (see ESM Methods). To identify a beta cell isomiR signature, we selected isomiR sequences with consistent expression across the four beta cell and human islet sequencing experiments, defined by >20 normalised counts and contributing at least 50% of the corresponding canonical miRNA expression (see ESM Methods).

### RNA-seq, quantification and analysis

For transcriptomic analysis using RNA-seq, 110 ng of RNA from human pancreatic islets, and 300 ng of RNA from the EndoC-βH1 human beta cell line, were first subjected to rRNA depletion using the NEBNext rRNA Depletion Kit v2 (New England Biolabs, Ipswich, MA, USA) following the manufacturer’s instructions. Then, the RNA obtained was purified using NEBNext RNA Sample Purification Beads, washed twice with 200 µl of 80% ethanol, and eluted with 7 µl of nuclease-free water.

Then, fragmentation and priming of rRNA-depleted RNA were performed by adding 4 µl of NEBNext First Strand Synthesis Reaction Buffer and 1 µl NEBNext Random Primers to 5 µl of eluted RNA. The reaction was incubated at 94°C for 15 min (as indicated for samples with RIN>7). After this, we performed first strand cDNA synthesis and second strand synthesis, then purifying the obtained cDNA using NEBNext Sample Purification Beads and eluted in 50 µl of 0.1X Tris-EDTA Buffer (provided in the kit). Then, end preparation and adapter ligation reactions were consecutively carried out on the purified cDNA. Finally, the ligated DNA was purified using NEBNext Sample Purification Beads and eluted in 20 µl of 0.1X TE Buffer and subjected to quality control using the Agilent 2100 Bioanalyzer to verify their profile and measuring DNA concentration through QUBIT 3.0 with the High Sensitivity DNA Kit. Libraries were normalised to 1.5 nmol/l, pooled, denatured and sequenced at a final concentration of 300 pmol/l on a NovaSeq 6000 platform.

FASTQ files from RNA-seq experiments were processed through a detailed bioinformatics workflow. Quality control was performed using FASTQC, followed by adapter trimming with fastp [55]. Trimmed reads were aligned to the human genome (GRCh38.p14) using the STAR algorithm [56], and PCR duplicates were removed with UMI-tools [57]. Transcript quantification at the exon level was conducted with featureCounts [58] (see ESM Methods). Low-expression genes were filtered out, and expression data were normalised using DESeq2’s median of ratios method. PCA was used to evaluate sample segregation across experimental conditions. Differential expression analysis identified a signature of genes consistently downregulated upon isomiR transfection, but not canonical miRNA transfection, compared with scrambled controls in both EndoC-βH1 and CI-HI samples. These genes, reflecting differences in seed sequence-mediated target binding, were validated computationally using TargetScan Custom (Version 5.2) and experimentally observed data, with overlaps assessed via hypergeometric test (see ESM Methods). The isomiR-specific target signature was then analysed for pathway enrichment using the Reactome Pathway Database (https://reactome.org/; accessed 4 Dec 2024) [59], identifying significantly regulated pathways (adjusted *p* value <0.05).

### Statistical analyses

Multiple linear regression was employed to evaluate associations between isomiRs expression and clinical parameters, adjusting for covariates such as age, sex and BMI. Influential data points were addressed using Cook’s distance to ensure robust model performance. For associations deemed significant (*p*<0.05), partial *R*^2^ was calculated to quantify and interpret effect size (see ESM Methods).

To validate transfection efficiency, differential expression of isomiR-411-5p-Ext5p(+1) and canonical miR-411-5p in transfected EndoC-βH1 cells and CI-HI was assessed. Small RNA-seq data were analysed using the DESeq2 Wald test, while ddPCR data were evaluated with a *t* test, comparing expression levels to scrambled controls (see ESM Methods).

Differential expression analysis of RNA-seq data was conducted using the DESeq2 Wald test, incorporating a paired design to account for variability stemming from cell passage or donor origin. Overlaps between isomiR-specific targets across datasets (EndoC-βH1 cells and CI-HI) or with computational predictions (TargetScan) were analysed using hypergeometric tests, validating the specificity of the isomiR-411-5p-Ext5p(+1) gene signature (see ESM Methods).

Insulin secretion and the stimulation index were compared across experimental conditions—EndoC-βH1 cells transfected with isomiR or canonical miR-411 vs scrambled sequences or not-transfected (nt)—using a Friedman test with Dunn’s multiple comparisons (see ESM Methods).

In all the experiments, randomisation and/or masking were not appropriate or not carried out for this study.

## Results

### IsomiRs represent a relevant fraction of human pancreatic islet and beta cell miRnome

IsomiRs are considered non-artefactual variants of miRNA sequences [9]. Consequently, due to their potential biological significance, a comprehensive characterisation of their expression profiles across various tissue and cellular contexts becomes of high importance. To analyse the abundance and distribution of isomiRs in human pancreatic islets and beta cells, we collected total RNA samples from LCM-HI from *n*=3 non-diabetic multiorgan donors and *n*=16 NGT living donors, and from *n*=3 different passages of the human beta cell line EndoC-βH1. Additionally, we analysed small RNA-seq datasets from CI-HI (*n*=3) and sorted beta cells (*n*=3) (ESM Fig. 1a). Sequencing data were processed and filtered with a state-of-the-art bioinformatic pipeline (see Methods and ESM Fig. 1b, c) to accurately quantify isomiRs and canonical miRNAs across the four datasets.

In LCM-HI we identified *n*=767 distinct sequences derived from *n*=164 miRNAs, including *n*=31 expressed exclusively as canonical miRNA, *n*=29 exclusively as isomiRs and *n*=104 as both canonical and isomiRs. In CI-HI we found *n*=1076 sequences from *n*=261 miRNAs, of which *n*=38 were expressed exclusively as canonical miRNAs, *n*=62 as isomiRs and *n*=161 as both. The sorted beta cell dataset showed *n*=1244 sequences from *n*=306 miRNAs, including *n*=34 canonical miRNAs, *n*=66 isomiRs and *n*=206 present as both. Finally, in the EndoC-βH1 cell line, *n*=914 sequences from *n*=243 miRNAs were identified, with *n*=45 expressed exclusively as canonical miRNAs, *n*=50 as isomiRs and *n*=148 as both (ESM Fig. 2a).

**Fig. 1:**
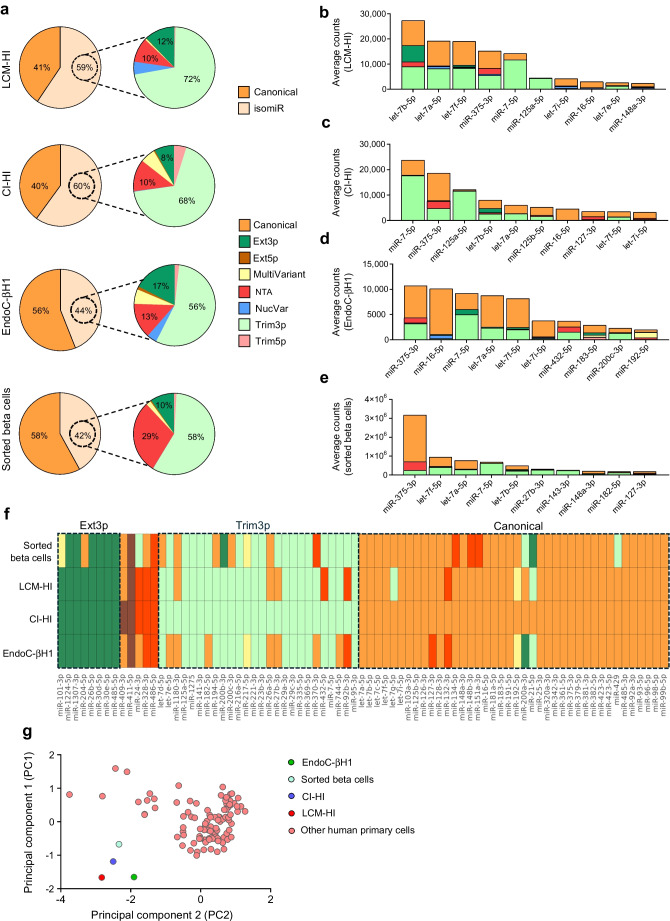
Analysis of isomiR and canonical miRNA profiles across different beta cell and islet samples. (**a**) Pie charts showing the distribution of isomiR and canonical miRNA forms in LCM-HI, CI-HI, EndoC-βH1 cells and sorted beta cells. The proportions of different isoforms are represented by different colours, as shown in the key. (**b**–**e**) Bar plots representing the average counts and composition of individual top-10 expressed miRNA species in LCM-HI (**b**), CI-HI (**c**), EndoC-βH1 cells (**d**) and sorted beta cells (**e**). The bars are colour coded to show the contributions of canonical and different isomiR forms. (**f**) Clustermap representing the group assigned to each of the *n*=79 miRNAs with consistent expression in the four experiments. The group was assigned according to the isomiR class with the higher contribution to its expression. The assignment of the prevalent isomiR classes to each miRNA is colour coded. (**g**) PCA of miRNA expression profiles performed on the average isomiR composition for the *n*=4 islet/beta cell datasets and the *n*=99 cell types retrieved from the isomiRdb repository. The PCA shows that the first *n*=2 principal components separate the beta cell dataset from the other cell types, suggesting a specificity in their post-transcriptional modifications. LCM-HI, CI-HI, sorted beta cells and EndoC-βH1 cells are marked to indicate clustering according to their miRNA profiles

In these datasets, isomiR/canonical expression revealed a high proportion of counts assigned to isomiRs in comparison with canonical miRNA sequences (LCM-HI: 59.2 ± 1.9%; CI-HI: 59.6 ± 2.4%; sorted beta cells: 42.3 ± 7.2%; EndoC-βH1: 43.8 ± 1.2%) (Fig. 1a) with Trim3p being the predominant modification. Additionally, a more in-depth analysis of isomiR expression was performed to describe different sequence variation types or classes. According to the modification, sequences were defined as 5′ or 3′ end trimming (Trim5p and Trim3p ), 5′ or 3′ end extension (Ext5p and Ext3p), NTA, MultiVariant, NucVar and canonical (see ESM Methods). Using this classification, we revealed a striking predominance of 3′ end modifications (Lv3p, including Trim3p , Ext3p and NTA), with Trim3p representing the largest isomiR fraction across all analysed datasets (LCM-HI: 71.8 ± 2.8%, CI-HI: 67.9 ± 6.5%, sorted beta cells: 57.8 ± 21.2% and EndoC-βH1 cells: 55.8 ± 1.0%) (Fig. 1a, Table 1). A detailed analysis of isomiR profiles across individual biological samples from each dataset revealed a high consistency in the isomiR/canonical ratio, isomiR class composition and isomiRs with seed sequence variations (nucleotide 2–7) (ESM Fig. 2b–d).

**Table 1:** IsomiR type fractions across the four human islet and beta cell datasets

IsomiR type	LCM-HI (% of total isomiRs)	EndoC-βH1 (% of total isomiRs)	CI-HI (% of total isomiRs)	Sorted beta cells (% of total isomiRs)
Ext3p	12.14 ± 1.85	17.40 ± 0.88	7.49 ± 0.84	9.69 ± 4.26
Ext5p	0.15 ± 0.04	1.21 ± 0.13	0.51 ± 0.19	0.48 ± 0.28
MultiVariant	0.60 ± 0.12	5.87 ± 0.61	5.60 ± 3.48	1.89 ± 1.33
NTA	9.82 ± 0.89	14.05 ± 1.22	12.70 ± 0.83	29.31 ± 17.13
NucVar	5.10 ± 1.32	3.96 ± 1.88	0.18 ± 0.09	0.07 ± 0.04
Trim3p	71.79 ± 2.78	55.79 ± 0.95	67.87 ± 6.51	57.76 ± 21.15
Trim5p	0.4 ± 0.11	1.72 ± 0.02	4.83 ± 2.89	0.88 ± 0.54

Data show average contribution (reported as percentage of total isomiR content) and the SD across samples

Next, we aimed to determine the contribution of isomiRs at the individual miRNA level. By analysing the top 10 most expressed miRNAs across the four human islet and beta cell datasets, we observed that isomiR composition varies markedly by individual miRNA (Fig. 1b–e and ESM Table 6). For example, the islet-enriched miR-375 exhibited a predominant canonical miRNA sequence expression (EndoC-βH1: 59.7 ± 2.4%; sorted beta cells: 77.3 ± 2.8%; CI-HI: 54.1 ± 9.0%; LCM-HI: 45.2 ± 2.0%) (Fig. 1b–e and ESM Table 6) while, in contrast, the beta cell enriched miR-7 showed a consistent and prevalent isomiR composition across all datasets, with Trim3p representing the major fraction (CI-HI: 72.2 ± 4.1%; LCM-HI: 82.4 ± 1.4%, sorted beta cells: 73.5 ± 12.2%; EndoC-βH1: 54.2 ± 2.0%) (Fig. 1b–e and ESM Tables 6–7).

To investigate whether miRNA post-transcriptional sequence processing is similar across islet and beta cell datasets, we focused on a subset of 79 miRNAs that exhibited consistent expression (ESM Fig. 2e) (see Methods and ESM Methods). Then, we analysed their isomiR composition patterns across the datasets, assigning each miRNA to a group based on the isomiR class that contributed most to its expression in each specific dataset. The resulting cluster map shows a strong consistency in the dominant isomiR class assigned to each miRNA (Fig. 1f, ESM Fig. 3a–d). Notably, we identified a cluster of *n*=26 miRNAs composed mainly by Trim3p isomiRs, *n*=8 miRNAs predominantly composed by Ext3p modifications and a cluster of *n*=40 miRNAs primarily expressed as canonical sequences; the remaining miRNAs (*n*=5) showed a tendency to be primarily expressed as NTA or Ext5p isomiRs (ESM Fig. 3a–d).

To determine whether the observed isomiR composition pattern is specific to islet and beta cell datasets or is common across multiple cell types and tissues, we analysed isomiRs from 396 publicly available sequencing datasets, representing 99 distinct human primary cell types from healthy donors, obtained from the isomiRdb repository (see Methods and ESM Methods). PCA focused on the isomiR composition pattern diversity revealed that islet and beta cell-enriched samples clustered together, while samples from other different 99 human primary cell types (not belonging to beta cells or islets) formed distinct, separate clusters (Fig. 1g). This segregation suggests that the isomiR composition patterns of human islets and beta cells reflect a distinctive profile, potentially shaped by cell-specific post-transcriptional modification mechanisms that influence the pool of expressed miRNAs.

### Beta cell isomiR signature is associated with beta cell function

**Fig. 2:**
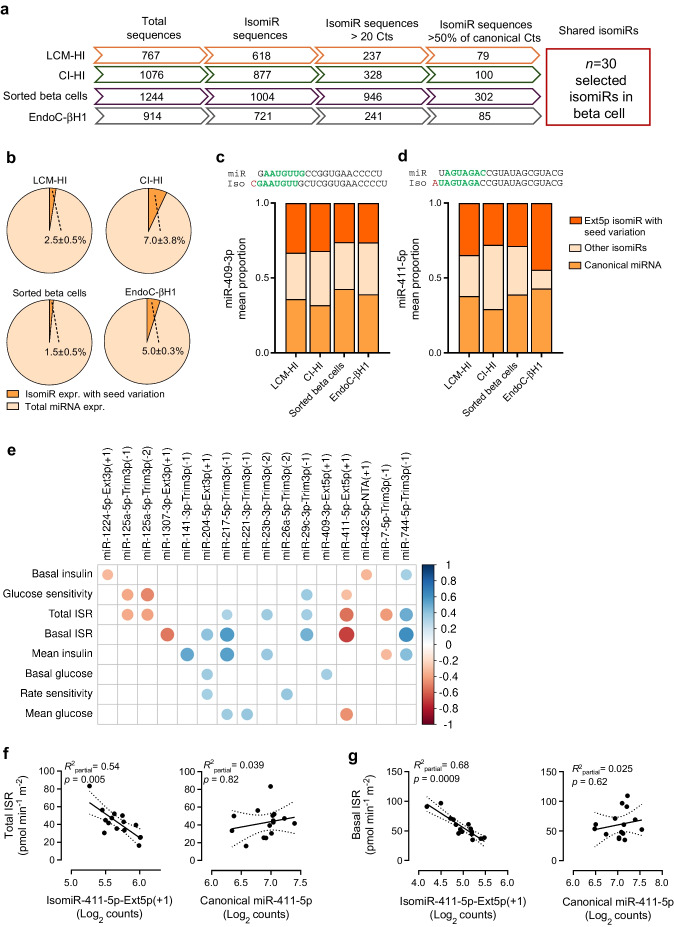
A beta cell isomiR signature is associated with functional parameters. (**a**) Workflow illustrating the filtering steps applied to identify a robust isomiR signature across human islet and beta cell datasets. The numbers indicate the number of sequences remaining after each step: (1) removal of canonical miRNAs, (2) exclusion of isomiRs with an average expression <20 normalised counts, (3) elimination of isomiRs contributing less than 50% to the expression of their corresponding canonical miRNAs. The final signature was derived from the intersection of the remaining isomiRs in the islet and beta cell datasets, ensuring reliability. (**b**) Pie charts showing the average contribution of isomiRs with seed variations to the total miRNA expression in islet and beta cell datasets. The percentages (mean ± SD) are colour coded to distinguish contributions of sequences with conserved and non-conserved seed sequences. (**c**–**d**) Bar plots showing the average expression composition of miR-409-3p (**c**) and miR-411-5p (**d**) with their canonical sequences, isomiR sequences with seed variation identified in the beta cell signature and other isomiR sequences across islet and beta cell datasets (the seed sequence is highlighted in green for the isomiR [Iso] and canonical miRNA [miR]). Each bar represents the mean contribution of the selected isomiR, the canonical sequence and other detected sequences for the miRNA, distinguished by different colours. (**e**) Correlation plot (corrplot) depicting significant associations between isomiRs and beta cell metabolic parameters (*p*<0.05). Associations were determined using multiple linear regression models, adjusted for age, sex and BMI. The square root of the partial *R*^2^ value, multiplied by the coefficient sign, is shown to indicate the strength and direction of the relationship. Colour intensity corresponds to the magnitude of the association, as indicated in the legend. The isomiR name is composed of the originating miRNA, the isomiR class and the number of added or trimmed nucleotides in brackets. (**f**, **g**) Scatter plots illustrating the association of isomiR-411-5p-Ext5p(+1) and its canonical miRNA counterpart with total ISR (**f**) and basal ISR (**g**). While the isomiR exhibits a significant correlation, no such association is observed for the canonical miRNA. The x-axis represents the log₂-transformed expression values, adjusted for covariates (age, sex and BMI). The *p* value for the clinical parameter’s coefficient and the partial *R*^2^ are provided for each plot. Cts, counts; expr., expression

To identify a set of specific isomiR sequences consistently detected across islet and beta cell datasets, we performed a comprehensive cross-validation and intersection analysis (Fig. 2a). This approach resulted in the identification of a signature of *n*=30 isomiR sequences (Fig. 2a and ESM Table 8), which may have a relevant functional role in islets and in beta cells. Given that the main prominent functional variation of isomiRs in relation to their canonical counterparts is due to modification in the nucleotide 2–7 seed sequence, we focused on isomiRs exhibiting such modification (i.e. Lv5p [Trim5p, Ext5p], NucVar or MultiVariant isomiRs).

The seed sequence conservation analysis revealed that only a small fraction of isomiRs showed seed changes (LCM-HI: 2.5 ± 0.5%, EndoC-βH1: 5.0 ± 0.3%; sorted beta cells: 1.5 ± 0.5%, CI-HI: 7.0 ± 3.8%) (Fig. 2b). However, among them, we identified two isomiRs contributing significantly to the overall miRNA expression across the four datasets: isomiR-411-5p-Ext5p(+1) and isomiR-409-3p-Ext5p(+1) (isomiR-411-5p-Ext5p(+1): LCM-HI: 34.8 ± 7.0%, EndoC-βH1: 44.6 ± 0.6%, CI-HI: 28.0 ± 10.9%, sorted beta cells: 28.7 ± 13.7%; isomiR-409-3p-Ext5p(+1): LCM-HI: 33.2 ± 5.6%, EndoC-βH1: 26.3 ± 2.8%, CI-HI: 32.0 ± 1.1%, sorted beta cells: 26.1 ± 12.8%) (Fig. 2c, d). The seed sequence changes observed for miR-411-5p (miR-411-5p: 5′-AGUAGAC-3′; isomiR-411-5p-Ext5p(+1): 5′-UAGUAGA-3′) and for miR-409-3p (miR-409-3p: 5′-AAUGUUG-3′, isomiR-409-3p-Ext5p(+1): 5′-GAAUGUU-3′) suggest differences of isomiR functions relative to the canonical counterparts.

In order to explore a potential function for these isomiRs in beta cells, we conducted a regression analysis adjusted for age, sex and BMI, between the *n*=30 isomiR expression signatures identified in LCM-HI and a set of parameters measured *in vivo* from the same NGT donors who were metabolically profiled (Fig. 2e). Remarkably, among the isomiRs displaying seed sequence changes, isomiR-411-5p-Ext5p(+1), but not its canonical counterpart, exhibited a major significant (*p*<0.05) inverse correlation with beta cell functional parameters, including basal ISR and total ISR (Fig. 2f and g). These results highlight the potential alternative functional role of isomiRs compared with their canonical counterparts and underline the importance of evaluating them and their alternative function.

### Overexpression of isomiR-411-5p-Ext5p(+1), but not its canonical, reduces GSIS in EndoC-βH1 cells

**Fig. 3:**
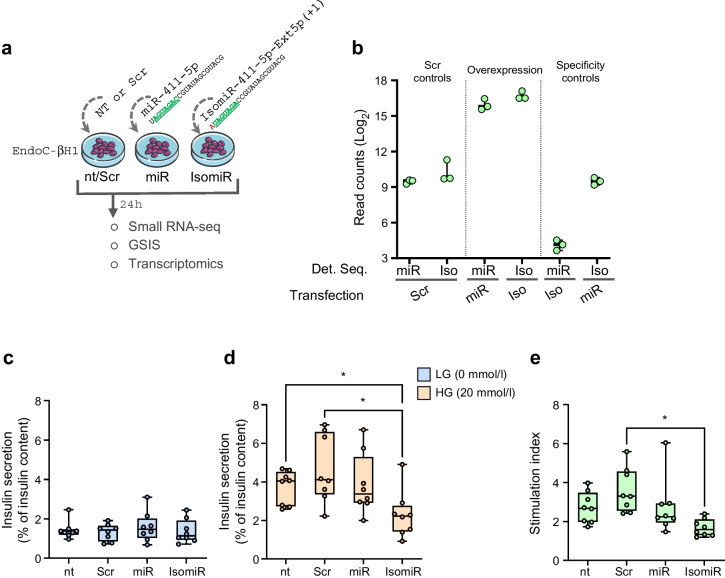
IsomiR-411-5p-Ext5p(+1) overexpression modulates GSIS in EndoC-βH1. (**a**) Schematic representation of the experimental workflow. EndoC-βH1 cells were transfected with either a scrambled control, miR-411-5p, or its isomiR [isomiR-411-5p-Ext5p(+1)] for 24 h. Following transfection, samples were analysed using small RNA-seq, GSIS and transcriptomics to assess functional and molecular changes. (**b**) Quantification of miRNA and isomiR expression levels in transfected EndoC-βH1 cells as determined by small RNA-seq. Read counts for detected sequences of miR-411-5p and isomiR-411-5p-Ext5p(+1) are presented on a log_2_ scale; *n*=3 independent experiments. (**c**, **d**) Insulin secretion was assessed in EndoC-βH1 cells not-transfected, transfected with scrambled control, miR-411-5p mimic or isomiR-411-5p-Ext5p(+1) mimic sequences and treated with (**c**) low glucose (0 mmol/l) or (**d**) high glucose (20 mmol/l). The boxplots report the median alongside min-to-max error bars of the percentage of insulin secreted over total insulin content. (**e**) Boxplot showing the median alongside min-to-max error bars of the stimulation index calculated as the ratio between high glucose (20 mmol/l) and low glucose (0 mmol/l) insulin secretion normalised for insulin content. *n*=8 independent experiments. Statistics using Friedman test with Dunn’s multiple comparison; *p<0.05. Det. Seq. detected sequences; HG, high glucose (20 mmol/l); Iso, isomiR-411-5p-Ext5p(+1)/isomiR-411 mimic; LG, low glucose (0 mmol/l); miR, miR-411-5p/miR-411 mimic; nt, not-transfected; Scr, scrambled control

To investigate the functional role of isomiR-411-5p-Ext5p(+1), we examined its effects on GSIS upon isomiR or canonical miRNA overexpression in EndoC-βH1 cells (Fig. 3a). First, we confirmed the specific overexpression of the isomiR or canonical miRNA, compared with scrambled and not-transfected (nt) controls, using small RNA-seq (Fig. 3b) and ddPCR analysis (ESM Fig. 4a–d).

Then, we measured insulin secretion both at low-glucose (0 mmol/l) and high-glucose (20 mmol/l) conditions. In EndoC-βH1 cells, the overexpression of isomiR-411-5p-Ext5p(+1) significantly decreased GSIS compared with scrambled and nt controls (mean ± SD: 51 ± 15.2%; *p*=0.01 and *p*=0.04, respectively) (Fig. 3d), while no significant changes were observed in low-glucose conditions (Fig. 3c); interestingly, the same effect was not observed upon overexpression of the canonical miR-411-5p (Fig. 3c–e). These findings suggest that isomiR-411-5p-Ext5p(+1), but not its canonical miRNA, exerts a glucose-dependent regulatory effect on insulin secretion in EndoC-βH1 cells, specifically inhibiting insulin release under stimulatory conditions.

### Divergent gene targeting by isomiR-411-5p-Ext5p(+1) and canonical miR-411-5p in beta cells

To elucidate the mechanisms specifically regulated by isomiR-411-5p-Ext5p(+1) in beta cells, we performed a genome-wide gene expression analysis using RNA-seq in EndoC-βH1 cells overexpressing isomiR-411-5p-Ext5p(+1) or canonical miR-411-5p.

**Fig. 4:**
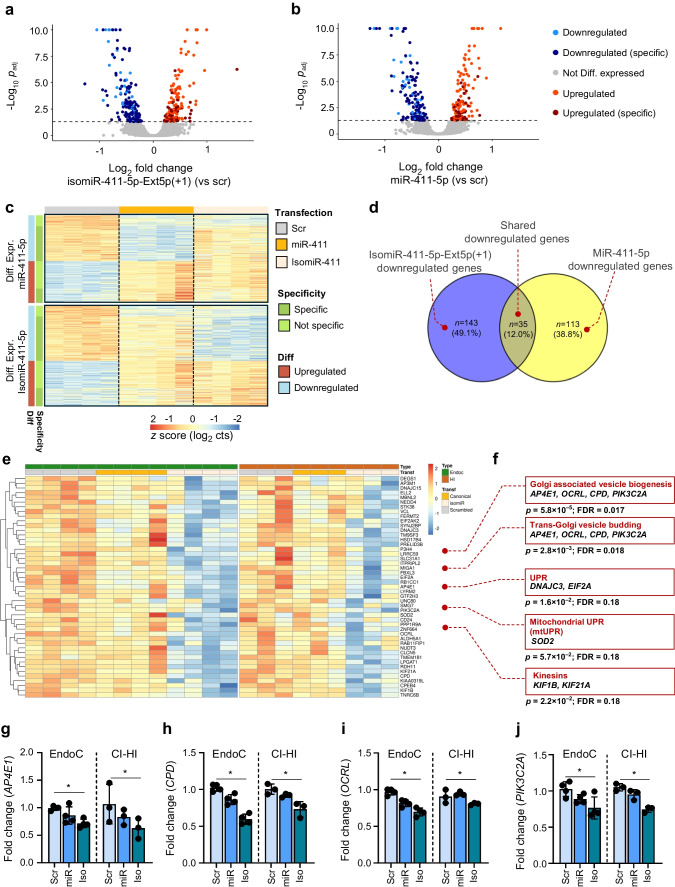
IsomiR-411-5p-Ext5p(+1) targets different genes compared with the canonical miRNA in EndoC-βH1 and CI-HI. (**a**, **b**) Volcano plots illustrating genes differentially expressed following transfection of isomiR (**a**) or canonical miRNA (**b**) compared with scrambled controls in the EndoC-βH1 cell line. The *x*-axis represents the log₂ fold change in expression relative to scrambled controls, while the *y*-axis shows the –log₁₀ adjusted *p* values. Genes are colour coded to indicate those regulated by both isomiR and canonical miRNA or specifically by either one. (**c**) Heatmap showing the *z* scores of log₂-scaled expression values for genes differentially expressed following isomiR-411-5p-Ext5p(+1) or canonical miR-411-5p transfection compared with scrambled controls. Genes are colour coded to indicate those regulated by both isomiR and canonical miRNA or specifically by either one. (**d**) Venn diagram showing the overlap of genes downregulated by isomiR-411-5p-Ext5p(+1) and canonical miR-4115p in EndoC-βH1 cells. The diagram indicates that the majority of genes downregulated by the isomiR are not affected by the canonical miRNA, with the number of genes and percentages reported. (**e**) Heatmap of the *z* scores for log₂-scaled expression values of the 47 genes specifically downregulated by isomiR-411-5p-Ext5p(+1) in both EndoC-βH1 cells and CI-HI. Hierarchical clustering was performed based on gene expression patterns, and gene names are listed on the right. (**f**) Reactome pathways enriched in genes specifically downregulated by isomiR-411-5p-Ext5p(+1). The pathway names, associated genes, *p* values and FDRs are shown. (**g**–**j**) Bar plots showing the fold change in expression for transcripts specifically downregulated by isomiR-411-5p-Ext5p(+1) in both EndoC-βH1 cells and CI-HI. Gene-specific downregulation is shown for *AP4E1* (**g**), *CPD* (**h**), *OCRL* (**i**) and PIK3C2A (**j**). Fold changes were calculated relative to scrambled-transfected controls, with a representative scrambled sample used as the reference to account for variability; *p<0.05. cts, counts; Diff. differentially/differential; EndoC, EndoC-βH1; Expr., expressed; Iso, isomiR-411-5p-Ext5p(+1)/isomiR-411 mimic; miR, miR-411-5p/miR-411 mimic; Scr, scrambled control

In total, *n*=13,158 expressed protein-coding genes were detected. The PCA showed no global separation of the samples by isomiR, canonical miRNA- and scrambled control-transfected samples, indicating that the transfection did not broadly alter the transcriptome (ESM Fig. 5a). Normalised data were then used as input for a differential expression analysis. Differential expression analysis was performed to compare samples transfected with the isomiR or canonical miRNA against scrambled control (DESeq2 Wald’s test, false discovery rate [FDR] <0.05). This analysis identified *n*=178 genes that were significantly downregulated and *n*=147 upregulated following isomiR transfection. In contrast, *n*=148 genes were downregulated and *n*=135 upregulated by the canonical miRNA (Fig. 4a, b). Of note, among differentially expressed genes we found a set of them specifically and exclusively modulated by isomiR-411-5p-Ext5p(+1) or by its canonical miRNA (Fig. 4c).

Interestingly, focusing on downregulated genes, potentially directly targeted by isomiR or canonical miRNA overexpression, only *n*=35 are commonly regulated by both, whereas *n*=143 are exclusively downregulated by the isomiR (Fig. 4c, d), while *n*=113 by the canonical miRNA. In contrast, we found *n*=88 upregulated genes that are shared between the isomiR and the canonical miRNA, while *n*=59 are uniquely upregulated by the isomiR and *n*=47 by the canonical miRNA (Fig. 4c).

To further validate the signature observed in EndoC-βH1 cells, RNA-seq was performed on CI-HI (*n*=3) transfected either with the canonical miR-411-5p, isomiR-411-5-Ext5p(+1) or scrambled control sequence. ddPCR analysis confirmed the specific overexpression of the canonical miR-411 or isomiR-411-5p-Ext5p(+1) compared with scrambled control (ESM Fig. 4e–h).

Transcriptomic analysis in islets revealed a total of *n*=12,911 expressed protein-coding genes. Of them, *n*=467 genes were significantly downregulated upon isomiR-411-5p-Ext5p(+1) overexpression (vs scrambled, DESeq2 Wald’s test, FDR <0.05) but not in miR-411-5p-transfected samples. Importantly, 47/467 downregulated genes were shared between CI-HI and EndoC-βH1 upon isomiR-411-5p-Ext5p(+1) overexpression (ESM Fig. 5b) but not differentially expressed in miR-411-5p samples (Fig. 4e). Statistical validation using a hypergeometric test (see Methods and ESM Methods) confirmed that this overlap was unlikely to occur by chance (*p*=2.4 × 10^−30^), emphasising the reliability of this signature.

Pathway analysis performed on the set of 47 genes specifically downregulated by isomiR-411-5p-Ext5p(+1) and shared between CI-HI and EndoC-βH1 (Fig. 4e), confirmed that isomiR-411-5p-Ext5p(+1) primarily modulates genes involved in insulin vesicles budding and trafficking (i.e. Golgi associated vesicle biogenesis [FDR=0.017] and trans-Golgi network vesicle budding [FDR=0.018]) (Fig. 4f and ESM Table 9) including *AP4E1*, *CPD*, *OCRL* and *PIK3C2A* (Fig. 4g–j). Additionally, other pathways central to beta cell function included unfolded protein response (UPR) or mitochondrial UPR (mtUPR) genes (*ElF2A* and *DNAJ3C*, *SOD2*) or kinesins (*KIF1B*, *KIF21A*) involved in granule trafficking. These results suggest an involvement of isomiR-411-5p-Ext5p(+1) in insulin processing and insulin granule trafficking, supporting the association of the isomiR expression with insulin secretion parameters in in vivo and in vitro contexts.

Finally, we verified whether the isomiR-specific downregulated genes included in the list of 47 genes, matched with the predictions from TargetScan. First, in silico target gene predictions confirmed the higher number of genes targeted by isomiR-411-5p-Ext5p(+1) in comparison with the canonical miRNA (*n*=110 vs *n*=64 genes). Second, 81/110 genes were predicted to be exclusively targeted by the isomiR (ESM Fig. 5c), confirming the partially divergent function between isomiR and canonical miRNA. Importantly 8/81 genes are part of the list of *n*=47 genes consistently downregulated by the isomiR (ESM Fig. 5d). This overlap was also validated by a hypergeometric test (*p*=1.1 × 10^−9^), further supporting the accuracy of the identified isomiR-specific gene signature and its strong alignment with seed sequence complementarity. Taken together these results demonstrate that isomiR-411-5p-Ext5p(+1) modulates central target genes involved in insulin granule trafficking and UPR response, independently of its canonical miRNA miR-411-5p.

## Discussion

IsomiRs are considered non-artefactual variants of miRNA sequences with specific functional roles in different contexts [9]. Notably, 5′ isomiRs feature a shifted seed sequence compared with canonical miRNAs, leading to significant modifications in their targetome [60]. Similarly, although less clear, 3′ isomiRs may have distinct non-canonical target recognition and, importantly, exhibit differences in half-life and/or AGO2 loading [61]. In both instances, isomiRs exhibit alternative functions compared with their canonical counterparts. Hence, in this context, the profiling of isomiRs can add a further layer of complexity, although improving our knowledge on islet and beta cell biology.

Extensive research has been focused on the investigation of isomiRs, particularly in relation to cancer [12, 62–67]. In the context of beta cells, just one study, in MIN6 mouse insulinoma cells [46], provided an overview of isomiR expression but did not deeply explore their functional role or systematically profile their composition.

In the present work, considering their increasingly important functional role, we conducted a comprehensive analysis of isomiRs in four different human islets and beta cell sources including microdissected and collagenase-isolated human pancreatic islets (CI-HI) from non-diabetic living or multiorgan donors, sorted human beta cell datasets, and the human beta cell line EndoC-βH1. We used a small RNA-seq approach, employing an up-to-date and reliable miRNA-seq library protocol, combined with a state-of-the-art bioinformatic pipeline for isomiR detection (sRNAbench) which was implemented with stringent multi-step filtering.

First, our findings reveal that isomiRs constitute a significant fraction of total miRNA expression in beta cells (up to 60%). A significant proportion of isomiRs consisted of 3′ end modifications with Trim3p as a prevalent component. Of note, 3′ end isomiR expression was not related to the overall RNA integrity variations in LCM-HI samples. Furthermore, analysis performed at the single-sample level highlights a high concordance of isomiR composition in samples from the same sequencing experiments, suggesting that sex-related differences are likely negligible. Interestingly, we observed a high concordance rate in terms of specific miRNAs showing a prevalent Trim3p fraction in all datasets, with 26 miRNAs displaying a highly similar composition pattern. This cluster includes miRNAs previously associated with significant functions in human islets and beta cells, such as the miR-29 family [68], miR-200 family [42] and miR-7a [43, 69]. Strikingly, we found that miR-7a, central to beta cell development and function, was primarily composed of Trim3p isomiRs, with some sequences trimmed by up to 4 nucleotides; this composition is consistent across all analysed datasets, confirming the presence of a conserved mechanism of post-transcriptional miRNA regulation in beta cells. Moreover, this unique pattern was not observed in other miRNAs, even those with higher expression (e.g. miR-375-3p or let-7b-5p). This suggests that the generation of Trim3p isomiRs may be highly regulated, sequence specific and not correlated with their expression levels.

Second, by intersecting and cross-validating the four islet and beta cell datasets, we obtained a pool of *n*=30 consistently expressed isomiR sequences contributing to at least 50% of canonical miRNA expression, thus potentially playing a key role in beta cell function. Among them, we identified two isomiRs presenting a 1-nucleotide extension at the 5′ end [isomiR-411-5p-Ext5p(+1), isomiR-409-3p-Ext5p(+1)] shifting their seed sequences, thus having relevant functional implications based on the changes in their targetome. Both isomiRs are equally expressed in relation to their canonical miRNAs or other sequences in both human islets and beta cells or EndoC-βH1 cells, suggesting that their biogenesis is an active mechanism in beta cells. The regression analysis between isomiR expression in biopsy-derived LCM-HI and in vivo metabolic clinical parameters measured on the same non-diabetic, NGT living participants, showed that isomiR-411-5p-Ext5p(+1) , but not its canonical miRNA (miR-411-5p), was negatively correlated with both basal ISR and total ISR. This observation, which is strictly related to a beta cell physiological context, suggests that isomiR-411-5p-Ext5p(+1) expression fluctuations could be involved in the modulation and/or fine-tuning of insulin secretory machinery. In the in vitro context, isomiR-411-5p-Ext5p(+1) overexpression was confirmed to modulate glucose-stimulated, but not basal, insulin secretion in EndoC-βH1 cells. Importantly, the same function was not observed for canonical miR-411-5p, suggesting a divergent function.

Third, in line with GSIS results, we observed a divergent targetome of isomiR-411-5p-Ext5p(+1) in comparison with its canonical miRNA. Indeed, TargetScan analysis of isomiR-411-5p-Ext5p(+1) and miR-411-5p target genes revealed 110 and 64 predicted targets, respectively, with only 29 genes shared between them. Moreover, the significant downregulation of a set of genes (*n*=47) specifically modulated by isomiR-411-5p-Ext5p(+1) but not by miR-411-5p overexpression, both in EndoC-βH1 cells and CI-HI, further confirmed their divergent functions. For this set of genes, the most significant pathways are those involved in vesicle biogenesis in the Golgi and trans-Golgi network (*AP4E1*, *CPD*, *OCRL*, *PIK3C2A*), vesicle trafficking (*KIF1B*, *KIF21A*) and UPR or stress response (*DNAJC3*, *ElF2A*, *SOD2*). The genes included in these pathways have been previously linked to insulin secretion and/or beta cell function. For instance, inhibition of phosphoinositide 3-kinase C2 alpha (PIK3C2A), a member of the phosphoinositide 3-kinase (PI3K) family, leads to decreased glucokinase expression in beta cells and reduced glucose, KCl or other secretagogues-stimulated insulin secretion in MIN6 and INS1 rodent beta cell lines [70, 71]. Carboxypeptidase D (CPD) also appears to play a compensatory role in insulin processing under high-glucose conditions, thus corroborating its involvement in insulin processing [72]. The kinesin family (including kinesin family member 1B [KIF1B] and 21A [KIF21A]) have previously been associated with insulin vesicle movement, thus supporting a key role in insulin secretion [73, 74]. Additionally the *SOD2* gene, encoding a key antioxidant mitochondrial, superoxide dismutase, has been reported to be central to maintaining low levels of oxidative stress [75], and thus proper protein folding [76], resulting in appropriate GSIS. Notably, *SOD2* deletion impairs insulin secretion [77].

Overall, isomiR-411-5p-Ext5p(+1) modulates GSIS by targeting multiple genes contributing to proper beta cell function while miR-411-5p shows a divergent function not connected to insulin secretion (ESM Table 9).

In conclusion, our study provides an unprecedented exploration of isomiR profiling in diverse beta cell contexts, ranging from primary human islets to sorted beta cells and cellular models. Our work advances the understanding of isomiRs by going beyond simple profiling to identify beta cell-specific regulatory networks and functional roles for these miRNA variants, providing a new framework for exploring isomiRs as key regulators of cellular processes. By identifying isomiR-411-5p-Ext5p(+1) as a critical modulator of beta cell function and linking its expression to insulin secretion and stress adaptation, we position isomiRs as important players in the intricate regulatory networks of beta cells. This mechanistic insight sets the foundation for future studies investigating isomiRs as biomarkers and therapeutic targets.

## Supplementary Information

Below is the link to the electronic supplementary material.

ESM (PDF 7521 KB)

## Data Availability

Raw and analysed data are available from the corresponding author upon request.

## Abbreviations

AGO2: Argonaute 2
ddPCR: Droplet digital PCR
Ext3p/5p: 3′/5′ end extension
GSIS: Glucose-stimulated insulin secretion
CI-HI: Collagenase-isolated human islets
IsomiR: Isoform of miRNA
ISR: Insulin secretion rate
LCM: Laser capture microdissection
LCM-HI: Laser capture microdissected human islets
Lv3p: 3′ length variant
Lv5p: 5′ length variant
MultiVariant: Multi-length variant
NGS: Next-generation sequencing
NGT: Normal glucose tolerant
NTA: Non-templated nucleotide addition
NucVar: Nucleotide variation
PCA: Principal components analysis
RIN: RNA integrity number
Trim3p/5p: 3′/5′ end trimming
UPR: Unfolded protein response
