# Supplementary material for: Comprehensive sequencing profile and functional analysis of IsomiRs in human pancreatic islets and beta cells
Source: Diabetologia. 2025 Mar 18;68(6):1261–78. doi: 10.1007/s00125-025-06397-4 (PMC12069488; doi:10.1007/s00125-025-06397-4)
Supplement: Supplementary file 1 — ESM (PDF 7521 KB) [file 125_2025_6397_MOESM1_ESM.pdf]

Electronic Supplementary Material (ESM)

ESM Methods

Study donors and human pancreas tissue collection

Human pancreatic sections analyzed in this study were obtained from pancreata of *n*=3 brain-dead adult non-diabetic multiorgan donors within the European Network for Pancreatic Organ Donors with Diabetes (EUnPOD) and from *n*=16 non-diabetic living donors undergoing pylorus-preserving pancreatoduodenectomy recruited at the Digestive Surgery Unit and studied at the Centre for Endocrine and Metabolic Diseases Unit (Agostino Gemelli University Hospital, Rome, Italy). Indications for surgery were periampullary tumors, pancreatic intraductal papillary tumors, mucinous cystic neoplasm of the pancreas, and nonfunctional pancreatic neuroendocrine tumors (**ESM Table 1**). Surgical pancreatic tissue specimens were snap frozen in liquid nitrogen and stored at -80°C embedded in Tissue-Tek OCT compound. The study protocol (ClinicalTrials.gov NCT02175459) was approved by the Ethical Committee Fondazione Policlinico Universitario Agostino Gemelli IRCCS – Università Cattolica del Sacro Cuore (P/656/CE2010 and 22573/14), and all participants provided written informed consent, followed by a comprehensive medical evaluation, as previously described [1] [2]. In INNODIA EUnPOD network, pancreata not suitable for organ transplantation were obtained with informed written consent by organ donors' next-of -kin and processed with the approval of the local ethics committee of the Pisa University (**ESM Table 2**). None of the patients enrolled had a family history of diabetes. Living donors patients underwent both a 75-g oral glucose tolerance test and glycated hemoglobin (HbA1c) testing to exclude diabetes, according to the American Diabetes Association criteria [3].

Laser capture microdissection of human pancreatic islets

Pancreatic human tissue samples *n*=19 from non-diabetic donors (**ESM Table 1** and **ESM Table 2**) were frozen in Tissue-Tek OCT compound and then 7-µm thick sections were cut from frozen OCT blocks. Sections were fixed in 70% Ethanol for 30 s, dehydrated in 100% Ethanol for 1 min, in 100% Ethanol for 1 min, in Xylene for 5 min and finally air-dried for 5 min. Laser Capture Microdissection (LCM) was performed using an Arcturus XT Laser-Capture Microdissection system (Arcturus Engineering, Mountain View, CA, USA) by melting thermoplastic films mounted on transparent LCM caps (cat. LCM0214 - ThermoFisher Scientific, Waltham, MA, USA) on specific islet areas. Intrinsic beta cells autofluorescence allowed the identification of human pancreatic islets for LCM procedure. Adhesive thermoplastic caps containing microdissected cells were incubated with 10 µl of Extraction Buffer (cat. kit0204 - ThermoFisher Scientific, Waltham, MA, USA) for 30 min at 42 ◦C and kept at −80°C until RNA extraction. Each microdissection was performed within 30 min from the staining procedure in a contamination-free-dehumidified environment with an external temperature of 16°C

to preserve RNA integrity. Overall, an endocrine mass of approximately $2x10^6$ μm^2 area for each case was collected for further molecular analysis. Endocrine cell enrichment in microdissected area was tested by RT-qPCR by analyzing INS (endocrine) and AMY1A (exocrine) genes expression as previously done [1].

Human Islets and sorted beta cells

Collagenase-isolated Human Islets (CI-HI) from non-diabetic organ donors were purchased from Tebu-Bio (Le Perray-en-Yvelines, France). Donors and batches characteristics are reported in **ESM Table 3**. Before shipment, purity and viability were assessed. After reception, human islets were cultured in RPMI1640 (1X) + GlutaMAX™ (cat.72400-021, ThermoFisher Scientific, Waltham, MA, USA) medium containing 5.5 mM glucose and supplemented with 10% FBS (cat. ECS0180L, EuroClone, Milan, Italy) 1% Antibiotic/Antimycotic (cat. A5955, Sigma Aldrich, St. Louis, MO, USA) and 1% L-glutamine (cat. G7513, Sigma Aldrich, St. Louis, MO, USA). After an overnight culture at 28°C and 5% CO2, the islets were dissociated in single cells by using Trypsin-EDTA solution (cat. T3924, Sigma Aldrich, St. Louis, MO, USA). Following dissociation and cell counting, the dissociated islet cells were plated at a seeding density of $1.5x10^5$ cells/well in 24-well plates and were transfected as reported in Cell transfection. Alternatively, human islets were obtained from the Cell isolation and Transplantation center of the University of Geneva through the Breakthrough T1D (former JDRF) award 31-2008-416 (ECIT Islets for Basic Research program). Human islets were cultured at 37°C and 5% CO_2 for few days in CMRL1066 medium supplemented with 10% fetal calf serum, 100U/mL penicillin and 100mg/mL streptomycin, 2mM L-glutamine and 10mM HEPES. FASTQ files from sorted beta cell small-RNA sequencing were downloaded from the SRA [7] database (accession PRJNA193453; samples SRR871671, SRR873401, SRR873410) [8]. Human islet samples were obtained from non-diabetic donors, and their purity was confirmed using dithizone labeling and qRT-PCR to quantify both endocrine (insulin, glucagon, and somatostatin) and exocrine (pancreatic lipase, pancreatic amylase, and chymotrypsin C) markers. For beta cell enrichment, n=3 samples of human islets underwent fluorescence-activated cell sorting (FACS) based on high zinc content, using Newport Green to isolate beta cells while excluding ductal and dead cells. Sorted beta cells were purified by selecting 7-AAD-negative, CA19-9-negative, and NG-bright populations [9]. The final purity of the FACS-enriched beta cell populations was between 90% and 95%. Only samples with a total RNA yield higher than 1 μg and high RNA quality (RIN > 7) were selected for library preparation and small RNA sequencing. Donor characteristics, sequencing instruments used, and library preparation protocols are detailed in **ESM Table 4**.

EndoC-βH1 cell culture

EndoC-βH1 human beta-cell line [6] was obtained by UniverCell-Biosolutions (Toulouse-France) and used between passages 74-88 for Small RNA seq and GSIS experiments and between passages

49-55 for RNA Seq experiment. EndoC-βH1 were cultured at 37 °C with 5% CO2 in coated flasks (coating medium: DMEM high-glucose cat. 51441C, Penicillin/Streptomycin 1% cat. P0781, ECM 1% cat. E1270 and Fibronectin from bovine plasma 0.2% cat. F1141 - all from Sigma Aldrich, St. Louis, MO, USA) and maintained in culture in low-glucose DMEM (cat. D6046) supplemented with 2% BSA fraction V (cat. 10775835001), β- Mercaptoethanol 50 μM (cat. M7522), L-Glutamine 1% (cat. G7513), Penicillin/Streptomycin 1% (cat. P0781), Nicotinamide 10 mM (cat. N0636), Transferrin 5.5 μg/mL (cat. T8158) and Sodium selenite 6.7 ng/mL (cat. S5261) (all from Sigma Aldrich, St. Louis, MO, USA).

RNA isolation and quality control

Total RNA was extracted from each LCM sample using PicoPure RNA isolation kit Arcturus (cat. kit0204 - ThermoFisher Scientific, Waltham, MA, USA) following manufacturer's procedure. Briefly, the cellular extracts were firstly mixed with 12.5 µl of 100% Ethanol to obtain an enrichment also in small RNAs and then transferred onto the purification column filter membrane. DNase treatment was performed using RNase-Free DNase Set (cat. 79254 - Qiagen, Hilden, Germany). Total RNA was finally eluted in 11 µl of Elution Buffer (DNase/RNase-Free Water). All LCM captures deriving from the same human sample were pooled and subjected to a subsequent concentration through Savant SpeedVac™ DNA130 centrifugal evaporator (ThermoFisher Scientific, Waltham, MA, USA). Total RNA was extracted from approximately 2.5×10^5 EndoC-βH1 and from 1.5×10^5 dispersed cells from Human Pancreatic Islets of non diabetic brain-dead donors through Direct-zol RNA Microprep Kit (cat. R2062-Zymo Research, Irvine, CA, US) following manufacturer's instructions. Briefly, the pelleted cells were resuspended in QIAzol (cat.79306, Qiagen, Hilden, Germany), mixed with equal volume of 100% Ethanol and transferred to Zymo-Spin™ IICR Column. DNase digestion was performed using RNase-Free DNase Set (cat. 79254, Qiagen, Hilden, Germany). Finally, RNA was eluted in 15 µl of DNase/RNase-Free Water.

In order to evaluate RNA abundance and purity, Agilent 2100 Bioanalyzer technology with RNA Pico chips (cat. 5067-1513 Agilent Technologies, Santa Clara, CA, USA) was performed for each RNA sample reporting RNA integrity number (RIN) and concentration and by excluding samples with RIN<5.0 (RIN and concentration values for each sample are shown in **ESM Table 5**).

Small-RNA seq

Small RNA seq for assessment of transfection was performed from 100 ng of input RNA using QiaSeq miRNA library kit (cat. 331505, QIAGEN, Hilden, Germany) and UMI indexes (cat. 331565, QIAGEN, Hilden, Germany), and obtained small RNA libraries were normalized to 4 nM and pooled to load a final concentration of 10 pM on MiSeq Illumina platform (Reagent Kit V3.0) and subjected to sequencing as previously reported [10]. For small RNA sequencing of LCM-HI and EndoC-βH1 cells, 1 ng of RNA input was used to generate Small RNA libraries, while 10 ng of RNA input were

used to generate Small RNA libraries using QiaSeq miRNA library kit (Qiagen, Hilden, Germany) following manufacturer's instructions; QiaSeq library kit chemistry assigns Unique Molecular Index (UMI) during reverse transcription step to every small RNA molecule, to enable unbiased and accurate small RNAome-wide quantification. cDNA from Small RNA libraries were amplified and purified using magnetic beads. Then, libraries quality control (QC) was performed measuring cDNA concentration through QUBIT 3.0 spectrofluorometer (Qubit™ dsDNA HS Assay Kit, ThermoFisher Scientific, Waltham, MA, USA) and assessing their quality using capillary electrophoresis in Bioanalyzer 2100 (Agilent High Sensitivity DNA kit cat. 5067-4626, Santa Clara, CA, US). High quality of libraries was evaluated considering electropherograms showing a peak comprised between 175 and 185 bp. Following libraries QC, all were normalized to 2 nM and pooled, denatured in 0.2 N NaOH and sequenced on Illumina NovaSeq 6000 platform (NovaSeq 6000 SP Reagent Kit v1.5 (100 cycles) using the XP protocol applying 75x1 single reads, or Illumina NextSeq550 platform (NextSeq 500/550 Mid Output Kit v2.5 (150 cycles), applying 75x1 single reads).

Cell transfection

In order to perform functional study of modulation by hsa-miR-411-5p and isomiR-411-5p-Ext5p(+1), EndoC-βH1 cells and dispersed Human pancreatic islets of non-diabetic brain-dead donors were plated at a density of respectively 2.5 and 1.5× 10^5 cells in 24-well plates. After respectively 24 and 48h, EndoC-βH1 cells and dispersed Human pancreatic islets were transfected with 5nM of miRIDIAN microRNA mimic sequences (Dharmacon, Lafayette, CO, USA): miRIDIAN microRNA Human hsa-miR-411-5p mimic (cat. C-300987-01-0020, seq. 5'-UAGUAGACCGUAUAGCGUACG-3'), custom isomiR-411-5p-Ext5p(+1) (seq. 5'(P)-AUAGUAGACCGUAUAGCGUACG-3') and 5 nM miRIDIAN microRNA Mimic Negative Control #1 and/or #2 (cat. CN-001000-01-20 and cat. CN-002000-01-20) using Lipofectamine RNAiMAX (cat. 13778-150, Invitrogen, Waltham, MA, US).

IsomiR quantification using ddPCR

Assessment of transfection was performed by performing Taqman advanced reverse transcription and miR-Amp (Life technologies, CA, USA) reactions on all the previously described samples (for both EndoC-βH1 and Human Islets) following manufacturer's instruction on 10 ng of extracted RNA. TaqMan Advanced miRNAs assays are particularly sensitive for the discrimination of nucleotide modifications at 5'-end of miRNA sequences. Then, we adopted these assays to distinguish between miR-411-5p and isomiR-411-5p-Ext5p(+1), differing each other by 1-nt at 5'end.

Briefly, both miR-411-5p (Taqman advanced assay cat. #A25576 ID478086_mir) and isomiR-411-5p-Ext5p(+1) (custom assay 5'-ATAGTAGACCGTATAGCGTACG-3') expression was evaluated through droplet digital PCR (ddPCR) detection.

In detail, ddPCR was performed on a BioRad QX200 system (BioRad, Mississauga, ON, Canada) using TaqMan probe assays (Life technologies, CA, USA). Each PCR reaction contained 11 μL of

QX200 Supermix, 1.7 µL of each 20X TaqMan advanced assay (miR or isomiR), 5.3 µL of H_2O and 4 µL of template cDNA in a final volume of 22 µL. The PCR reactions were mixed, centrifuged briefly and 20 µL transferred into the sample well of a DG8 cartridge. After adding 70 µL of QX200 droplet generation oil into the oil wells, the cartridge was covered using a DG8 gasket, and droplets were generated using the QX200 droplet generator. Droplets were carefully transferred into PCR plates using a multi-channel pipette and the plate was sealed using PCR plate heat seal foil and the PX1 PCR plate sealer. PCR was carried out using a SimpliAmp thermal cycler (Life Technologies, CA, USA). The protocol included an initial denaturation at 95°C for 10 minutes, followed by 45 cycles of denaturation at 95°C for 30 seconds and annealing at the optimal temperature of 62°C. The process concluded with a final extension at 98°C for 10 minutes and a hold at 4°C for 30 minutes. After amplification, PCR plates were transferred to a QX200 droplet reader to quantify positive and negative droplets. Thresholds to separate positive from negative droplets were set manually for each miRNA using the histogram function and reads analyzed using QuantaSoft Analysis Pro software (Version 1.2, BioRad, Mississauga, ON, Canada).

GSIS and ELISA assay

For GSIS measurement, EndoC-βH1 cells were seeded in 12-well plates at a seeding density of 3.75 x 10^5 cells per well. The cells were incubated at 37°C in humidified air and 5 % CO2 and transfected with negative control, miR-411-5p and isomiR-411-5p-Ext5p(+1) sequence as reported in *Cell transfection*. After the transfection, growing medium was changed to starvation medium ULTI-ST (Human Cell Design, Toulouse, France) and incubated O/N. The day after, the starving medium was removed and β-KREBS (Human Cell Design, Toulouse, France) supplemented with fraction V BSA (cat. 10775835001, Sigma Aldrich, St. Louis, MO, USA), was added, and cells were pre-incubated for 1 h and then replaced with β-KREBS-BSA supplemented with 0 mmol/l glucose (no glucose added) or 20 mmmol/l glucose for 40 min. After the stimulation, the medium was collected and centrifuged at 700 x g for 5 minutes at 4°C; the resulting supernatant was recovered and stored at -20°C for up to four weeks before measuring the amount of secreted insulin. For intracellular insulin, lysis solution (1 ml 1 M Tris (pH 8.0), 500 µl Triton X-100, 5 ml Glycerol, 1.37 ml 5 M NaCl, 500 µl 0.2 M EGTA, 41.63 ml H2O with 1x protease inhibitor cocktail tablet (Roche complete mini EDTA-free per 10ml) was added to each well and centrifuged at 700 x g for 5 minutes at 4°C. Samples were stored at -20 °C for up to four weeks before measuring retained insulin. Insulin was determined by using a human insulin Enzyme-Linked Immunosorbent Assay (ELISA) Kit (cat. 10-1113-01, Mercodia, Uppsala, Sweden). ELISA was carried out following the manufacturers' instructions (cat. 10-1113-01, Mercodia, Uppsala, Sweden). Insulin values for both secreted and retained insulin were then calculated using a standard curve derived from known standards provided by the kit (cat. 10-1113-01, Mercodia, Uppsala, Sweden).

Bioinformatic pipeline for isomiR analysis and classification

To allow an accurate isomiR quantification and classification, a state-of art bioinformatic pipeline was implemented (**Fig S2a**). FastQ files obtained from the sequencing were analyzed with the sRNAbench online pipeline [11]. Reads were pre-processed in accordance with the library preparation protocol to remove adapters and, where applicable, PCR duplicates. Given the focus on isomiR analysis, a stringent quality control step was implemented to ensure high base-level accuracy across all reads. Using the minimum quality score threshold per sequence nucleotide, all reads with at least 1 nucleotide with a quality score (Q) < 30 (Phread+33) were discarded from the analyses. Reads were mapped in genome mapping mode, using the Human reference Genome Reference Consortium Human Build 38 patch release 13 (GRCh38.p13) [12]. Alignment was performed with the bowtie algorithm using the seed option with length (L) = 20, maximum number of mismatches (N) = 1, minimum length of the read =15, minimum number of read count =2 and maximum number of multiple mappings equal to 10. The seed alignment options of bowtie allow a maximum of N (1) mismatches in the first L (20) nucleotides, thus accounting for the great variability affecting the 3' end of miRNAs. Mapped reads were annotated to miRNAs (and isomiRs) using miRBase release 22.1 [13]. After the quantification step and removal of low-quality reads the raw isomiRs counts matrix was obtained from the "*microRNAannotation*" file of each sample. Sequences assigned to multiple miRNAs (usually resulting from Trim3p) were removed from the counts matrix. Once removed ambiguous sequences, the next filtering step was aimed to remove sequences that are likely to be sequencing errors. This second filtering step, referred to as relative abundance filter, occurred after the transformation of raw reads counts in reads per million (RPM), to account for differences in library size. Read counts were converted into RPM using the *cpm* function from the EdgeR package (version 3.40.2) [14]. RPM for each miRNA (resulting from the canonical sequence and its isomiR sequences) were summed up, and the contribution of each sequence to the total miRNA's RPM was estimated. Sequences whose average contribution (relative abundance) across samples to total miRNA expression was < 1% were removed from the analysis. To evaluate the goodness of the relative abundance filtering in removing possible sequencing errors from the analysis, an in-silico simulation was performed (See *In-Silico Simulation*). Alongside the relative abundance filtering, specific low-count filters are applied to the dataset to remove sequences with low expression. After filtering, read counts were normalized using DESeq2's median of ratios method (v1.38.3) [15] (to control for library size and RNA composition differences). IsomiRs were then classified according to their *isoLabel* based on the sRNAbench classification. In detail, *n*=6 different main classes (or types) of isomiRs were identified, with some of them that can be divided into further subclasses:

- Canonical: if the read perfectly corresponds to the reference sequence on miRbase v22.1.

- 3'length variant (Lv3p): a length variant that affects the 3′ end of a miRNA. This class of modification includes both the loss of nucleotides (trimming, Trim3p) and the addition of nucleotides concordant with the precursor sequence (extension, Ext3p)
- 5'length variant (Lv5p): a length variant that affects the 5′ end of a miRNA. This class of modification includes both the loss of nucleotides (trimming, Trim5p) and the addition of nucleotides concordant with the precursor sequence (extension, Ext5p)
- Non-templated nucleotide addition (NTA): the addition of nucleotides at the 3′ end of a miRNA such as by terminal nucleotidyl transferases (TENTs).
- Multi-length variant (MultiVariant): a length variant that affects both ends of a miRNA sequence.
- Nucleotide variant (NucVar): an internal sequence variation as introduced by enzymes such as ADARs.

Being isomiRs classification based on a hierarchy, sequences with different modifications can be assigned to the same class (i.e., sequences with both trimming and nucleotide variation will be assigned to the trimming class). For this reason, to detect isomiRs with a different seed sequence compared to the canonical counterpart, a seed conservation analysis was performed. For each isomiR, nucleotides in position 2-7 were identified as the seed sequence. The same procedure was performed for canonical miRNAs, according to miRbase reference sequence. Then, isomiRs' seed sequences were compared to the seeds of their canonical counterparts. If differences in the sequences were detected, the isomiR was determined as 'Not-conserved seed' isomiR.

In-Silico Simulation

To evaluate the goodness of the relative abundance filtering in removing possible sequencing errors from the analysis, an in-silico simulation was performed using a string of *n*=22 elements (synth_seq), representing the canonical miRNA. The sequencing of the synth_seq was simulated with a probability of error in the reading of each nucleotide equal to 0.1% (Q=30). To model the error in the reading of each nucleotide with Q=30, a vector of *n*=1,000 elements, with *n*=999 equal to False and *n*=1 equal to True was defined (error_vect). Thus, the probability to sample an element from error_vect equal to True (1/1,000) represents the probability of an error in the sequencing of the synth_seq. The sequencing was simulated with the following procedure:

(i) For each nucleotide of the synth_seq, an element of error_vect was randomly sampled. (ii) If the sampled element was equal to False, the corresponding nucleotide of the sequencing simulation was set as equal to the nucleotide of the synth_seq, otherwise it was randomly selected a different nucleotide. This step resulted in the creation of a simulated read (referred to as synth_read), which could be equal or not to the original synth_seq. This 2-steps procedure was repeated *n*=1.000.000 times, thus originating an equal number of synth_reads (referred to as synth_pool). This pool of synth_reads was used for the validation of the relative abundance threshold. First, the occurrences

(counts) and the relative abundance of each sequence from the synth_pool were estimated. Only sequences with 1 single sequencing error were used to model the probability distribution of false isomiRs. Indeed, sequences with more sequencing errors have few counts and, consequently, a very low relative abundance. This is because (i) there is a very low chance of having multiple sequencing errors and (ii) there is a high number of possible combinations. For this reason, a threshold on the relative abundance capable of removing false isomiRs with 1 single error will certainly remove sequences with multiple errors. The false isomiRs relative abundance distribution was estimated using a normal distribution. The goodness of the relative abundance threshold was computed as the probability of having an isomiR with a relative abundance >1%, given the previously estimated false isomiRs relative abundance distribution. Among the reads originated from the sequencing' simulation, *n*=978,011 were equal to synth_seq. On the other hand, the *n*=21,989 reads detected as false isomiRs were assigned to *n*=274 different sequences. Among them, *n*=21,773 false isomiR reads (>99%) belonged to the *n*=66 sequences with a single nucleotide variation compared to the synth_seq. The counts assigned to the single nucleotide variation sequences ranged from 298 to 377 counts. The relative abundance of these sequences to the total miRNA counts was estimated as the counts assigned to the sequence divided by the total number of reads (1,000,000). The Shapiro test assessed the normality of the distribution of their relative abundance (p=0.23). Thus, the probability distribution of false isomiRs was modelled with a normal distribution with the mean and standard deviation of their relative abundance (mean=0.000330, sd= 0,000017) (Fig S2b). The probability of false isomiRs with a relative abundance higher than the threshold (0.01) was estimated with the pnorm function (p=0). The simulation corroborates the importance of the implementation of a filtering step based on the contribution to the corresponding miRNA counts. Indeed, false isomiRs with >300 read counts were detected. These false isomiRs are likely to be kept from the low counts filtering. Thus, miRNAs with very high levels of expression (i.e., miR-16-5p) could give rise to sequencing errors that will be kept in the further analyses if no filters based on relative abundance are implemented.

Small-RNA seq data analysis

FASTQ files from the four beta cell and human islets (HI) sequencing experiments (*n*=19 samples of LCM-HI, *n*=3 samples of CI-HI, *n*=3 samples of sorted beta cells, and *n*=3 independent passages of the EndoC-βH1 cell line) and from the pilot experiment to evaluate the transfection of the isomiR-411-5p-Ext5p(+1) /Canonical miR-411-5p in EndoC-βH1 (*n*=3 Ctr1, *n*=3 Ctr2, *n*=3 isomiR-411-5p-Ext5p(+1) transfected and= 3 canonical miR-411-5p transfected) were analyzed with the previously described bioinformatic pipeline. Reads from the LCM-HI, CI-HI, and EndoC-βH1 experiments were pre-processed following the Qiagen protocol (with UMIs) for adapter trimming and duplicate removal. Due to different library protocols, FASTQ files from the sorted beta-cell experiment required distinct processing methods. For samples SRR873401 and SRR873410, adapter trimming was performed

using the Illumina TruSeq library protocol. For the sample SRR871671, adapter sequences were manually identified and removed with Cutadapt (v1.18) [16]. The identified adapter sequence, "*ATCTCGTATGCCGTCTTCTGCTTG*," was trimmed with parameters set to a maximum error rate of 10% (-*e 0.1*), a minimum overlap of 3 (-*O 3*), and a minimum read length of 10 (-*m 10*). The resulting adapter-trimmed FASTQ file was then analyzed with sRNAbench as pre-trimmed data.

Specific low-count filters were defined for each dataset according to sequencing depth and sample size in order to remove sequences with low expression. The thresholds were set as follows:

- EndoC-βH1 (*n*=3 biological replicates): ≥ 3 counts in 66% of the samples
- LCM-HI (*n*=19 biological replicates): ≥ 3 counts in 68% of the samples
- Sorted beta cells (*n*=3 biological replicates): ≥ 3 counts per million in 66% of the samples
- CI-HI (*n*=3 biological replicates): ≥ 3 counts per million 66% of the samples
- EndoC-βH1 transfection study (*n*=12 biological replicates): ≥ 5 counts per million in 20% of the samples

Filtered counts matrices were then normalized with DESeq2's median of ratios method to account for differences in library size. The composition of each miRNA in the *n*=4 beta cell and human islets datasets was computed as the percentage of normalized counts assigned to the different isomiR classes, averaged across samples.

To evaluate the consistency and specificity of miRNAs post-transcriptional modifications in beta-cells, a subset of miRNAs with robust expression across all sequencing experiments was selected, thereby excluding low-expression miRNAs that could contribute to noise, and then compared to online reported datasets from multiple human primary cells sources.

Specifically, miRNAs (canonical sequence + isomiR sequences) with an average expression above 20 normalized counts in all sequencing experiments were included. Once a subset of reliable miRNAs consistently expressed in beta cells was identified, the average proportion of each isomiR class for each miRNA was computed across the different datasets. Each miRNA was then assigned to the isomiR class contributing the most to its total expression, resulting in a matrix associating each miRNA in each experiment with its dominant isomiR class. The matrix was visualized using the *pheatmap* function (Version 1.0.12) to assess the consistency of isomiRs class membership for the different miRNAs across the four sequencing experiments. Overall, we classified *n*=79 different miRNAs showing a consistent isomiRs type classification across the four different sequencing datasets.

To determine whether these miRNAs post-transcriptional modification fingerprints are specific to beta cells, isomiR expression data from different cell types were obtained from the isomiRdb repository [17]. Datasets from isomiRdb were downloaded and re-analyzed to evaluate the specificity of the post-transcriptional modifications in beta cells. Sample metadata, along with isomiR and canonical miRNA expression data (in Reads Per Million), were retrieved from isomiRdb and re-analyzed using a multi-step filtering process. First, metadata was used to select sequencing data

derived from healthy, primary cell studies. Only sources from the Sequence Read Archive (SRA) that included cell origin information were retained. The remaining dataset was then cross-referenced using external IDs to collect study titles and attributes from NCBI (https://www.ncbi.nlm.nih.gov/). Additional filters were applied at this stage, resulting in a final metadata set containing data from *n*=396 sequencing samples across *n*=99 distinct cell types. Following this, isomiR expression data were filtered according to the previously defined sample metadata, and the mean expression of each sequence for each cell type was calculated. The same relative abundance filtering applied in the earlier isomiR analyses was also implemented on the data from isomiRdb repository, to ensure consistency and reliability. After obtaining a consistent and reliable matrix of isomiR expression data, containing the average expression of each isomiR and canonical miRNA sequence across different cell types, sequences from isomiRdb were classified into isomiR classes based on the previous definition. Since isomiRdb data lacked the "*isoLabel*" annotation, direct classification into isomiR classes was not possible. Therefore, classification was performed only for sequences with an *isoLabel* annotation available in the sRNAbench files from the previous four beta cells and HI sequencing experiments. This approach allowed us to classify 99.9% of *isomiRdb* expression data for the *n*=79 consistently expressed miRNAs in beta cells and HI sequencing experiments. Once an isomiR class was assigned to the isomiRdb sequences from other cell types, the composition of each miRNA per cell type (the percentage of expression assigned to the previously defined isomiR classes) was calculated. The composition of each of the previously identified consistently expressed miRNAs was then used as input for a Principal Component Analysis (PCA). This PCA included cell types from both isomiRdb and the Beta-cell enriched Sequencing Experiments, with the aim of evaluating whether beta cell-containing samples could be separated from other cell types based on their isomiR profiles.

To identify a beta cell isomiRs signature, we assumed that if an isomiR sequence is consistently expressed across the four beta cells and HI sequencing experiments, it is likely expressed in beta cells and may potentially play a real functional role. To achieve this, we applied a series of filtering criteria to select isomiR sequences that show strong, consistent expression. Specifically, we included only those isomiR sequences that (i) represented at least 50% of the corresponding canonical miRNA counts on average, and (ii) had an average expression level exceeding 20 normalized counts across all the *n*=4 beta cell and HI sequencing experiments.

RNA seq, quantification and analysis

Total RNA samples were depleted of ribosomal RNA using the NEBNext rRNA Depletion Kit v2 (Human/Mouse/Rat; New England Biolabs, NEB #E7400, #E7405). This kit employs an RNase H-based workflow that removes both cytoplasmic and mitochondrial rRNA, enriching non-ribosomal RNA content. This step increases the detection of less abundant transcripts, enhancing the biological relevance of transcriptomic analyses.

For RNA from CI-HI, 110 ng, and for RNA from the EndoC-βH1 human beta cell line, 300 ng, were added to a hybridization mixture composed of 2 μL of NEBNext v2 rRNA Depletion Solution and 2 μL of NEBNext Probe Hybridization Buffer. The reaction was incubated in a preheated thermal cycler at 105°C for 15–20 minutes, briefly spun down, and placed on ice to halt the reaction.

RNase H digestion was performed by preparing a mix of 2 μL RNase H Reaction Buffer, 2 μL NEBNext Thermostable RNase H, and 1 μL nuclease-free water. After adding 5 μL of this digestion mix to the hybridized RNA, the mixture was incubated at 50°C for 30 minutes in a thermal cycler.

DNA contamination was removed by DNase I digestion. A mix containing 5 μL DNase I Reaction Buffer, 2.5 μL NEBNext DNase I, and 22.5 μL nuclease-free water was added to the RNase H-treated RNA and incubated at 37°C for 30 minutes. RNA was purified using NEBNext RNA Sample Purification Beads, vortexed for resuspension. A volume of 90 μL was added to the DNase I-treated RNA and incubated on ice for 15 minutes. The beads were separated using a magnetic rack, washed twice with 200 μL of 80% ethanol, and eluted with 7 μL of nuclease-free water.

Fragmentation and priming of rRNA-depleted RNA were performed by adding 4 μL of NEBNext First Strand Synthesis Reaction Buffer and 1 μL NEBNext Random Primers to 5 μL of eluted RNA. The reaction was incubated at 94°C for 15 minutes. First strand cDNA synthesis followed, with 20 μL of a reaction mixture (8 μL NEBNext Strand Specificity Reagent and 2 μL NEBNext First Strand Synthesis Enzyme) added to the primed RNA. The reaction proceeded at 25°C for 10 minutes, 42°C for 15 minutes, 70°C for 15 minutes, and held at 4°C.

Second strand synthesis was carried out by adding 80 μL of a mix (8 μL NEBNext Second Strand Synthesis Reaction Buffer, 4 μL NEBNext Second Strand Synthesis Enzyme Mix, and 48 μL nuclease-free water) to the first strand cDNA synthesis reaction and incubating at 16°C for 1 hour. Double-stranded cDNA was purified using NEBNext Sample Purification Beads and eluted in 50 μL of 0.1X TE Buffer.

End preparation of the cDNA was performed by adding 60 μL of an end prep mix (7 μL NEBNext Ultra II End Prep Reaction Buffer and 3 μL NEBNext Ultra II End Prep Enzyme Mix) to the purified cDNA. The mixture was incubated at 20°C for 30 minutes, then 65°C for 30 minutes, and held at 4°C. Adapter ligation followed, with a reaction mixture containing 5 μL NEBNext Unique Dual Index UMI RNA Adapter, 1 μL NEBNext Ligation Enhancer, and 30 μL NEBNext Ultra II Ligation Master Mix added to the end-prepped cDNA.

The ligated DNA was purified using NEBNext Sample Purification Beads and eluted in 20 μL of 0.1X TE Buffer. DNA library quality was assessed using the Agilent 2100 Bioanalyzer and considered for sequencing if showing a bump profile with a mean peak of circa 300 bp. DNA concentration was measured using QUBIT 3.0 with the High Sensitivity DNA Kit. Libraries were normalized to 1.5 nM, pooled, denatured, and sequenced using the NovaSeq 6000 platform. FASTQ files from the paired-end RNA sequencing were obtained using the bcl-convert command line tool (Version 4.0.3). Quality control of these FASTQ files was performed with the FastQC tool (Version 0.12.1) to assess overall

sequencing quality, and results were aggregated using MultiQC [18] (Version 1.16). The fastp tool [19] (Version 0.23.4) was subsequently implemented to trim remaining adapters. Trimmed FASTQ files were then aligned to the human genome using the STAR algorithm [20] (Version 2.7.10b) with the Genome Reference Consortium Human Build 38 patch release 14 [12] (GRCh38.p14) as reference. STAR alignment settings included paired-end mode, a mismatch tolerance (*outFilterMismatchNoverReadLmax*) of 4%, a minimum intron length (*alignIntronMin*) of 20, a maximum of 20 multi-mappings per read (*outFilterMultimapNmax*), and a genomic sequence length around annotated junctions (*sjdbOverhang*) of 75. The resulting BAM files were indexed with Samtools (Version 1.11). PCR duplicates were removed from these files using the UMI-tools algorithm [21] (Version 1.1.2), which was configured in paired-end mode to enhance deduplication and filter out sequences with low mapping quality (*Quality < 10*). Transcript quantification was performed with the featureCounts algorithm [22] (Version 2.0.1), utilizing the GRCh38.p14 GTF annotation file as a reference. Settings included fragment counting (-p) for paired-end sequencing, exonic region mapping (-t ‘exon’), and reverse-stranded read setting (-s 2). This pipeline produced a raw counts matrix indicating the number of deduplicated paired-reads aligned to exonic regions.

To evaluate samples segregation based on experimental condition (transfection with isomiR, Canonical, or Scrambled) within each experimental setup, a Principal Components Analysis (PCA) on RNA-seq raw counts matrix was performed. The DESeq2 [15] (Version 1.38.3) package's variance stabilizing transformation was applied to the raw counts matrix to achieve homoscedastic data for PCA analysis, which was then performed on centered data, preserving gene-specific variability by not scaling the data. Following PCA, low-expression genes were filtered out of the raw counts matrices for EndoC-βH1 and CI-HI samples. Counts were scaled to Counts Per Million (CPM) using the EdgeR package [14] (Version 3.40.2) to standardize for library size differences across samples. Transcripts with at least 5 CPM in two or more samples were retained for further analyses, while others were excluded. Filtered counts matrices were then normalized with DESeq2’s median of ratios method to account for differences in library size and RNA composition, producing normalized counts matrices. The resulting normalized counts matrices were subsequently used in differential expression analysis to identify genes showing significant differences in expression across experimental conditions (**See Statistical Analyses**).

Transcripts showing significant downregulation upon isomiR transfection, with no significant change following canonical miRNA transfection were identified as specific targets of the isomiR. This specificity likely arises due to differences in the seed region between isomiR and canonical miRNA, which influence target-binding affinity. Results from both CI-HI and EndoC-βH1 cells were merged to identify a comprehensive gene signature of transcripts consistently downregulated by isomiR across both cellular models, reflecting the isomiR’s unique regulatory effect in both primary and cellular models. The significance of the overlap between isomiR-specific targets in CI-HI and EndoC-βH1 was assessed using a hypergeometric test (See Statistical Analyses). This signature was then

used as input for an enrichment analysis with the Reactome Pathway Database [23], to identify pathways significantly regulated by the signature. Pathways with adjusted $p<0.05$ were detected as significantly regulated. To further validate the gene signature, a computational approach using TargetScan Custom [24] (release 5.2) was employed. Seed sequences of the isomiR and canonical miRNA were provided to predict targets based on conserved 8mer and 7mer binding sites. Genes predicted as targets for the isomiR, but not for the canonical miRNA, were identified as putative specific targets. The overlap between computationally predicted and experimentally observed isomiR-specific targets was then evaluated using another hypergeometric test (See Statistical Analyses)

Statistical analyses

To evaluate the association between the expression of isomiRs from the signature and beta-cell functional and metabolic parameters from the LCM-HI cohort, multiple linear regression analyses were performed. First, normalized read counts for each isomiR were transformed to log2 scale, with a pseudo count added to avoid log-transformation issues at zero counts. In the regression models, the log2-transformed isomiR expression was set as the dependent variable, while the clinical parameter, the age, the sex, and the BMI were implemented as independent variables. To identify and exclude potential influential data points, Cook's distance was calculated for each point within each model. Data points with a Cook’s distance exceeding 5 times the average Cook’s distance of the model were considered influential and removed, reducing the risk of single data points disproportionately affecting the regression outcomes. After this adjustment, linear models were estimated individually for each isomiR in relation to each clinical parameter. Models in which the p associated with the clinical parameter coefficient < 0.05 were considered as statistically significant, regardless of the effects of the covariates (age, sex, and BMI). For significant associations, the partial R^2 of the clinical parameter was estimated using the *partial_r2* function from the sensemakr package [25] (Version 0.1.6). The partial R^2 was then multiplied by the sign of the clinical parameter's coefficient, to reflect both the strength and the direction of the association. For visualization purposes, the log2-transformed isomiR counts were adjusted to account for covariates effect in each regression. This adjustment was achieved by calculating the covariate effect for each data point as the product of the covariate’s coefficient and its value, summing these covariate effects, and then subtracting this total from the normalized log2-scaled counts. This method effectively isolates and removes the overall influence of covariates on isomiR expression, offering a clearer view of the clinical parameter's association with isomiR expression.

To evaluate the differential expression of isomiR-411-5p-Ext5p(+1) and canonical miR-411-5p sequences in the EndoC-βH1 transfected cells, and thereby assess transfection efficiency, DESeq2 Wald’s test was applied to normalized small-RNA sequencing read counts. Expression levels in cells transfected with isomiR-411-5p-Ext5p(+1) or canonical miR-411-5p were compared to scrambled

controls, with *p* adjusted using the Benjamini-Hochberg method. To further validate transfection efficiency in both EndoC-βH1 as well as in CI-HI, TaqMan Advanced miRNA chemistry ddPCR data were log2 scaled, and a t-test, with *p* adjusted using the Benjamini-Hochberg method, was employed to analyze the differential concentration of isomiR-411-5p-Ext5p(+1) and canonical miR-411-5p. Concentrations in transfected EndoC-βH1 and CI-HI cells were compared to scrambled controls.

To assess the significance of differences in insulin secretion and Stimulation Index following transfection with isomiR-411-5p-Ext5p(+1) or canonical miR-411 sequences, a Friedman test was conducted, followed by Dunn's multiple comparison post hoc analysis. This approach compared EndoC-βH1 cells transfected with isomiR-411-5p-Ext5p(+1) and canonical miR-411 to those transfected with control mimics and scrambled sequences, providing a thorough evaluation of how each transfection condition impacted insulin secretion and the Stimulation Index.

To perform differential expression analysis in the RNA-sequencing experiment, a design that accounts for the variability related to the paired setup was implemented. This setup included EndoC-βH1 samples from identical passages and CI-HI samples from identical donors, each transfected with Canonical miRNA, isomiR, or scrambled. The design integrated both the transfection condition (Canonical, IsomiR, or Scrambled) and the pairing variable (Cell-line passage or Donor), enabling the identification of condition-specific expression differences while controlling for variability stemming from cell passage or donor origin. Differential expression was evaluated by comparing samples transfected with isomiR against those transfected with scrambled and samples transfected with Canonical miRNA against scrambled. DESeq2 Wald's test was used to test the differential expression and resulting *p* were adjusted using the Benjamini-Hochberg method to correct for multiple comparisons. Transcripts with $padj < 0.05$ were classified as differentially expressed between conditions.

To assess the significance of the overlap between the signature of isomiR-411-5p-Ext5p(+1)-specific targets in CI-HI and EndoC-βH1, a hypergeometric test was used. This test calculates the probability of observing a given overlap between the two sets by chance, based on the following parameters:

- q the number of genes identified as isomiR-411-5p-Ext5p(+1)-specific target both in EndoC-βH1 and CI-HI
- m the total number of isomiR-411-5p-Ext5p(+1) specific targets in EndoC-βH1
- n the total number of genes in the dataset not identified as isomiR-411-5p-Ext5p(+1)-specific targets in the EndoC-βH1 (i.e., the number of genes detected in both EndoC-βH1 and CI-HI, minus m)
- k the total number of isomiR-411-5p-Ext5p(+1)-specific targets in CI-HI.

The hypergeometric cumulative distribution function (phyper*(q , m, n, k, lower.tail=F))* was used to calculate the probability of observing at least *q* overlapping genes by chance, providing statistical validation of the specificity of the isomiR-411-5p-Ext5p(+1) gene signature.

To evaluate the overlap between computationally predicted (from TargetScan) and experimentally observed isomiR-specific targets, another hypergeometric test was used, with the following parameters:

- q the number of genes identified as both predicted and experimentally observed isomiR-specific targets
- m the total number of experimentally observed isomiR-specific targets
- n be the total number of genes in the dataset not identified as experimentally observed isomiR-specific targets (i.e., the number of genes detected in both EndoC-βH1 and CI-HI, minus m)
- k be the number of genes predicted by TargetScan as isomiR-specific targets.

The probability of observing at least *q* overlapping genes by chance was calculated (*phyper(q , m, n, k, lower.tail*=*F)*), offering further evidence of the biological relevance and specificity of the isomiR-411 gene signature.

ESM Tables

ESM Table 1. Values are reported as mean and standard deviation (except for sex) of demographic and clinical-metabolic characteristics of non-diabetic living donors (*n*=16).

**Clinical**	**Value**
Age (years)	65.12±10.22
BMI(kg/m2)	25.13±3.65
Sex (M/F)	8/8
basal ISR (pmol.min^{-1}.m^{-2})	60.12±22.21
basal glu (mmol/l or mM)	5.06±0.55
basal ins (pmol/l)	39.45±18.76
glu sens (pmol.min^{-1}m^{-2}.mM^{-1})	87.61±36.09
mean glu (mmol/l or mM)	7.63±1.01
mean ins (pmol/l)	301.81±126.99
rate sens (nmol.m^{-2}.mM^{-1})	889.07±928.21
total ISR (pmol.min^{-1}.m^{-2})	42.18±15.59

ESM Table 2. Characteristics of non-diabetic multiorgan donors (*n*=3) recruited within INNODIA EUnPOD network.

Case ID	Sex (M/F)	Age (y)	BMI (kg/m^2)	AutoAb (ELISA)	HiRes HLA	Cause of death
LCM-1	M	39	23.6	GADA negative, IA-2A negative, ZnT8A negative	HLA:A*03,33; B*14, B*14 SD B65; C*08; DRB1*01, DQB1*05	Trauma
LCM-2	M	49	25.8	GADA negative, IA-2A negative, ZnT8A negative	HLA:A*03,68; B*35,47; C*04,06; DRB1*03,08; DQB1*02,04	Cardiovascular disease
LCM-3	F	46	32.5	GADA negative, IA-2A negative, ZnT8A negative	HLA:A*24; B*15,18; C*7; DRB1*04,11; DQB1*3	Cardiovascular disease

ESM Table 3. Characteristics of brain-dead non diabetic donors (*n*=6) and islet batches quality indicators.

**Distributor**	**Donor ID**	**Age (y)**	**Sex (M/F)**	**BMI (kg/m^2)**	**HbA1c (%)**	**Cause of death**	**N° of IEQ**	**Purity (%)**	**Islet viability (%)**
TebuBio (Prodo Labs)	HP-24024-01	56	F	32.4	5.8	Stroke	5,000	90	95
TebuBio (Prodo Labs)	HP-24067-01	55	M	29.9	5.6	Anoxic Event	10,000	90	95
TebuBio (Prodo Labs)	HP-24151-01	38	M	23.2	5.7	Anoxic Event	10,000	90	95
ECIT Center (University of Geneva)	HI9	59	F	23.7	n/a	Cerebral trauma	15,000	84	90
ECIT Center (University of Geneva)	HI12	47	F	33.4	n/a	Cerebral haemorrage	30,000	88	90
ECIT Center (University of Geneva)	HI7	54	M	28.5	n/a	Cerebral trauma	30,000	60	89

ESM Table 4. Table showing the characteristics of non-diabetic pancreatic donors from whom beta cells were sorted (SRA accession number)

**SRA accession number**	**Sex (M/F)**	**Age (y)**	**BMI (Kg/m2)**	**Cause of Death**	**Instrument**	**Library Protocol**
SRR871671	M	41	29	Meningioma	GAIIx	Illumina v1.5
SRR873401	F	51	31	NA	HiSeq 2000	TruSeq v1
SRR873410	F	59	29	NA	HiSeq 2000	TruSeq v1

ESM Table 5. Table showing RNA integrity Number (RIN, scale 0-10) and concentration (pg/µl) of total RNA extracted from LCM islets captured from pancreatic tissue sections of non-diabetic subjects and EndoC-βH1 samples.

Case ID	RNA Integrity Number (RIN) value	Concentration (pg/µl)
LCM-1	6.1	905
LCM-2	6	271
LCM-3	7.9	285
LCM-4	5.9	326
LCM-5	6	292
LCM-6	5.8	348
LCM-7	7.5	195
LCM-8	6.3	990
LCM-9	6.5	231
LCM-10	7.1	489
LCM-11	5.6	369
LCM-12	7.3	193
LCM-13	7.2	599
LCM-14	6	391
LCM-15	5.9	310
LCM-16	7.2	236
LCM-17	6.8	349
LCM-18	7.5	660
LCM-19	5.2	1,085
ENDOC-1	8.5	995
ENDOC-2	9.3	1,000
ENDOC-3	8.5	1,000

ESM Table 6. IsomiRs composition of the *n*=10 most expressed miRNAs in the four Human islets and beta cell datasets. The average proportion (percentage) and its standard deviation across samples are reported. MiRNAs are listed from the most expressed to the least expressed. Only isomiRs classes represented in at least one miRNA are reported for each dataset.

LCM-HI(*n*=19)

matureName	Ext3p	NTA	Trim3p	canonical	NucVar
hsa-let-7b-5p	23.7±2.2 %	7.2±0.5 %	32.9±3.2 %	36.3±1.5 %	0
hsa-let-7a-5p	1.4±0.2 %	0	43±2.3 %	51.4±2.1 %	4.2±1.1 %
hsa-let-7f-5p	3.4±0.5 %	1.4±0.1 %	44±3.8 %	49.6±3.8 %	1.5±0.5 %
hsa-miR-375-3p	0	15.4±1.2 %	36±3.3 %	45.3±2 %	3.3±0.9 %
hsa-miR-7-5p	0	0	82.4±1.4 %	17.6±1.4 %	0
hsa-miR-125a-5p	0	1.5±0.4 %	96.7±0.7 %	1.7±0.4 %	0
hsa-let-7i-5p	0	6.7±1 %	8.3±1 %	67.9±4 %	17.1±4.5 %
hsa-miR-16-5p	1.2±0.3 %	0	4.7±1 %	79.5±3.5 %	14.6±3.7 %
hsa-let-7e-5p	3.9±0.9 %	3.8±0.5 %	47.7±2.3 %	43.1±2.2 %	1.4±0.4 %
hsa-miR-148a-3p	2±0.4 %	12±1.3 %	13.6±2 %	57.6±3.2 %	14.7±4.3 %

CI-HI (*n*=3)

matureName	Ext3p	Trim3p	canonical	MultiVariant	NTA	Trim5p
hsa-miR-7-5p	1.1±0.1 %	72.2±4.1 %	26.7±4.2 %	0	0	0
hsa-miR-375-3p	0	22.6±8.6 %	54.1±9 %	1.4±1.1 %	13.9±2.1 %	8.1±7.8 %
hsa-miR-125a-5p	0	94.8±1.3 %	3.8±1.1 %	0	1.5±0.3 %	0
hsa-let-7b-5p	21.4±5.7 %	31.1±7.2 %	41±2.1 %	0	6.4±1.8 %	0
hsa-let-7a-5p	1.5±0.4 %	42.5±3.6 %	56.1±3.2 %	0	0	0
hsa-miR-125b-5p	0	31±4.7 %	60.7±4.4 %	0	8.3±0.7 %	0
hsa-miR-16-5p	1.3±0.5 %	1.3±0.3 %	90±6.9 %	0	0	7.5±7.6 %
hsa-miR-127-3p	0	9.4±2.4 %	57.5±8.8 %	0	33.1±10.4 %	0
hsa-let-7f-5p	2.9±2.1 %	38.5±5.5 %	57±6.5 %	0	1.6±0.7 %	0
hsa-let-7i-5p	0	10.8±3.5 %	73.8±8.2 %	0	15.3±4.8 %	0

EndoC-βH1 (*n*=3)

matureName	NTA	NucVar	Trim3p	canonical	Ext3p	MultiVariant	Trim5p
hsa-miR-375-3p	9.5±0.7 %	1.5±0.5 %	29.3±2 %	59.7±2.4 %	0	0	0
hsa-miR-16-5p	0	6.3±3.2 %	2.1±0.3 %	89.6±2.5 %	2±0.5 %	0	0
hsa-miR-7-5p	0	0	54.2±2 %	34.7±2.2 %	11.1±0.3 %	0	0
hsa-let-7a-5p	0	0	26.2±0.2 %	71.3±0.2 %	2.5±0.1 %	0	0
hsa-let-7f-5p	1.3±0.2 %	0	24.3±0.7 %	70.2±0.8 %	4.2±0 %	0	0
hsa-let-7i-5p	4.8±0.4 %	4.8±2.6 %	6.1±0.7 %	84.3±1.8 %	0	0	0
hsa-miR-432-5p	27.9±3.4 %	0	40.7±2.3 %	31.4±1.1 %	0	0	0
hsa-miR-183-5p	0	0	2.1±0.5 %	52.3±0.7 %	15.3±1 %	13.8±1 %	16.5±1.1 %
hsa-miR-200c-3p	6.2±0.3 %	0	55.1±0.7 %	38.7±0.7 %	0	0	0
hsa-miR-192-5p	16.2±1 %	0	1.5±0.3 %	23.8±0.6 %	3.7±0.9 %	52.6±2.2 %	2.3±0.1 %

Sorted beta cells (*n*=3)

matureName	NTA	Trim3p	canonical	Ext3p	Trim5p
hsa-miR-375-3p	16±8.3 %	6.7±5.6 %	77.3±2.8 %	0	0
hsa-let-7f-5p	4.8±1 %	37.9±5.2 %	53.6±2.5 %	3.8±2.2 %	0
hsa-let-7a-5p	6.4±2.5 %	31.5±3.6 %	60.3±1.9 %	1.8±0.2 %	0
hsa-miR-7-5p	0	73.5±12.2 %	24.8±13.6 %	0	1.7±1.5 %
hsa-let-7b-5p	7.4±2 %	33.5±7.8 %	46.9±4.5 %	12.1±1.7 %	0
hsa-miR-27b-3p	3.8±1.1 %	71.5±18.9 %	22.4±17.7 %	2.2±1.2 %	0
hsa-miR-143-3p	12.4±8.6 %	77.5±17.1 %	10.1±9.2 %	0	0
hsa-miR-148a-3p	6.1±1.5 %	1.3±0.6 %	67±18.6 %	25.6±17.9 %	0
hsa-miR-182-5p	1.9±1.4 %	62.2±1.8 %	32.4±1.3 %	3.5±1.4 %	0
hsa-miR-127-3p	21.1±8.5 %	37.1±36.1 %	41.8±27.7 %	0	0

ESM Table 7. Table showing the relative expression (in percentage) of different sequences assigned to miR-7 in beta-cell and human islet datasets. The table includes the following information: the sequence, the isomiR class (indicating the number of nucleotides added or removed), and the mean and standard deviation of expression percentages across the datasets. The seed sequence is highlighted in green, while any nucleotide variation is marked in red. At the bottom of the table, the three precursor sequences of miR-7 are shown, with the canonical miRNA sequence underlined.

Sequence	Class	LCM-HI	Sorted beta cells	CI-HI	EndoC-βH1
TGGAAGACTAGTGATTTTGTTGTT	Canonical	17.6 ±1.4 %	24.8±13.6 %	26.7±4.2 %	34.7±2.2 %
TGGAAGACTAGTGATTTTGTTGTTG	Ext 3p(+1)	0	0	1.1±0.1 %	4.6±0.2 %
TGGAAGACTAGTGATTTTGTTGTTT	Ext 3p(+1)	0	0	0	4.1±0.1 %
TGGAAGACTAGTGATTTTGTTGTTGT	Ext 3p(+2)	0	0	0	2.4±0.2 %
CGGAAGACTAGTGATTTTGTTGT	Trim 3p(-1) (+NucVar)	1.4±0.4 %	0	0	0
TGGAAGACTAGTGATTTTGCTGT	Trim3p(-1) (+NucVar)	1.5±0.4 %	0	0	0
TGGAAGACTAGTGATTTTGTTGT	Trim3p (-1)	50.5±1.9 %	46.7±2.3 %	50.5±2.6 %	41.8±1 %
TGGAAGACTAGTGGTTTTGTTGT	Trim3p(-1) (+NucVar)	1.4±0.4 %	0	0	0
TGGAAGGCTAGTGATTTTGTTGT	Trim3p(-1) (+NucVar Seed)	1.9±0.5 %	0	0	0
TGGAGGACTAGTGATTTTGTTGT	Trim3p(-1) (+NucVar Seed)	2.3±0.6 %	0	0	0
TGGAAGACTAGTGATTTTGTTG	Trim3p(-2)	15.9±1.4 %	12.1±8.2 %	16.1±2.6 %	8.2±0.9 %
TGGAAGACTAGTGATTTTGTT	Trim3p(-3)	5.6±1.2 %	8.2±1.6 %	4.4±0.3 %	4.2±0.3 %
TGGAAGACTAGTGATTTTGT	Trim3p(-4)	1.8±0.6 %	6.5±4.6 %	1.2±0.1 %	0
CTAGTGATTTTGTTGTT	Trim5p(-7)	0	1.7±1.5 %	0	0

5'--- GTTCTGTGTGGAAGACTAGTGATTTTGTTGTTTTAGATAACTA **---3'** hsa-miR-7-1 precursor
5'--- GGCCCCATCTGGAAGACTAGTGATTTTGTTGTTGTCTTACTGCGC **---3'** hsa-miR-7-2 precursor
5'--- GTGCTGTGTGGAAGACTAGTGATTTTGTTGTTCTGATGTACTAC **---3'** hsa-miR-7-3 precursor

ESM Table 8. Table showing the signature of beta-cell isomiRs, including the sequence of each isomiR, the originating miRNA, and the isomiR class indicating the number of nucleotides added or removed

sequence	miRNA	Class
ACTCGGCGTGGCGTCGGTCGTGG	hsa-miR-1307-3p	Ext3p(+1)
AGAGGTAGTAGGTTGCATAGT	hsa-let-7d-5p	Trim3p(-1)
AGCTACATTGTCTGCTGGGTTT	hsa-miR-221-3p	Trim3p(-1)
ATAGTAGACCGTATAGCGTACG	hsa-miR-411-5p	Ext5p(+1)
ATCACATTGCCAGGGATTACC	hsa-miR-23b-3p	Trim3p(-2)
CGAATGTTGCTCGGTGAACCCCT	hsa-miR-409-3p	Ext5p(+1)
GTGAGGACTCGGGAGGTGGA	hsa-miR-1224-5p	Ext3p(+1)
GTGAGGACTCGGGAGGTGGAG	hsa-miR-1224-5p	Ext3p(+2)
GTGGGGGAGAGGCTGT	hsa-miR-1275	Trim3p(-1)
TAACACTGTCTGGTAAAGATG	hsa-miR-141-3p	Trim3p(-1)
TAACACTGTCTGGTAACGATGTT	hsa-miR-200a-3p	Ext3p(+1)
TAACAGTCTACAGCCATGGTCGT	hsa-miR-132-3p	NTA(+1)
TAATACTGCCGGGTAATGATGG	hsa-miR-200c-3p	Trim3p(-1)
TAATACTGCCTGGTAATGATG	hsa-miR-200b-3p	Trim3p(-1)
TACTGCATCAGGAACTGATTGG	hsa-miR-217-5p	Trim3p(-1)
TAGCACCATTTGAAATCGGTT	hsa-miR-29c-3p	Trim3p(-1)
TCAAGAGCAATAACGAAAAATG	hsa-miR-335-5p	Trim3p(-1)
TCCCTGAGACCCTTTAACCTGT	hsa-miR-125a-5p	Trim3p(-2)
TCCCTGAGACCCTTTAACCTGTG	hsa-miR-125a-5p	Trim3p(-1)
TCTTGGAGTAGGTCATTGGGT	hsa-miR-432-5p	Trim3p(-2)
TCTTGGAGTAGGTCATTGGGTGT	hsa-miR-432-5p	NTA(+1)
TGCGGGGCTAGGGCTAACAGC	hsa-miR-744-5p	Trim3p(-1)
TGGAAGACTAGTGATTTTGTTGT	hsa-miR-7-5p	Trim3p(-1)
TGGCTCAGTTCAGCAGGAACAGT	hsa-miR-24-3p	NTA(+1)
TGTAAACATCCCCGACTGGAAGCT	hsa-miR-30d-5p	Ext3p(+2)
TGTAAACATCCTTGACTGGAAGCT	hsa-miR-30e-5p	Ext3p(+2)
TTCAACGGGTATTTATTGAGC	hsa-miR-95-3p	Trim3p(-1)
TTCAAGTAATCCAGGATAGG	hsa-miR-26a-5p	Trim3p(-2)
TTCAAGTAATTCAGGATAGGTT	hsa-miR-26b-5p	Ext3p(+1)
TTCCCTTTGTCATCCTATGCCTG	hsa-miR-204-5p	Ext3p(+1)

ESM Table 9. Table showing results from pathway enrichment analysis performed using the Reactome database on the signature of 47 genes specifically downregulated by isomiR-411-5p-Ext5p(+1) in both EndoC-βH1 cells and CI-HI. The table includes the pathway name, the number of genes from the signature detected in the pathway, the total number of genes in the pathway, the ratio of genes found, and the *p* and FDR associated with the pathway enrichment.

Pathway name	Found	Total	Ratio	p	FDR
Golgi Associated Vesicle Biogenesis	4	61	0.00	0.00	0.02
trans-Golgi Network Vesicle Budding	4	80	0.01	0.00	0.02
Antiviral mechanism by IFN-stimulated genes	4	172	0.01	0.00	0.18
PKR-mediated signaling	3	88	0.01	0.00	0.18
Deregulated CDK5 triggers multiple neurodegenerative pathways in Alzheimer's disease models	2	30	0.00	0.00	0.18
Neurodegenerative Diseases	2	30	0.00	0.00	0.18
Synthesis of PIPs at the Golgi membrane	2	32	0.00	0.01	0.18
Mitochondrial unfolded protein response (UPRmt)	2	33	0.00	0.01	0.18
Defective Intrinsic Pathway for Apoptosis	2	33	0.00	0.01	0.18
Inhibition of PKR	1	2	0.00	0.01	0.19
Stimuli-sensing channels	3	121	0.01	0.01	0.19
Association of TriC/CCT with target proteins during biosynthesis	2	40	0.00	0.01	0.19
Interferon Signaling	5	397	0.03	0.01	0.19
FOXO-mediated transcription of oxidative stress, metabolic and neuronal genes	2	49	0.00	0.01	0.19
Defective visual phototransduction due to RDH12 loss of function	1	4	0.00	0.01	0.19
The canonical retinoid cycle in rods (twilight vision)	2	53	0.00	0.01	0.19
Unfolded Protein Response (UPR)	3	156	0.01	0.02	0.19
Phospholipid metabolism	4	314	0.02	0.02	0.19
Kinesins	2	68	0.00	0.02	0.19
Synthesis of PIPs at the plasma membrane	2	69	0.00	0.02	0.19
Post-transcriptional silencing by small RNAs	1	7	0.00	0.02	0.19
Membrane Trafficking	6	669	0.04	0.03	0.19
Gene and protein expression by JAK-STAT signaling after Interleukin-12 stimulation	2	73	0.00	0.03	0.19
TYSND1 cleaves peroxisomal proteins	1	8	0.00	0.03	0.19
ISG15 antiviral mechanism	2	83	0.01	0.03	0.19
Interleukin-12 signaling	2	84	0.01	0.03	0.19
Recycling of eIF2:GDP	1	10	0.00	0.03	0.19
Regulation of PTEN localization	1	10	0.00	0.03	0.19
Ion channel transport	3	209	0.01	0.03	0.19
Regulation of NPAS4 mRNA translation	1	11	0.00	0.04	0.19
Downregulation of ERBB4 signaling	1	11	0.00	0.04	0.19
Inositol phosphate metabolism	2	90	0.01	0.04	0.19
Degradation of GABA	1	12	0.00	0.04	0.19
SUMOylation of immune response proteins	1	12	0.00	0.04	0.19
Cellular response to mitochondrial stress	1	12	0.00	0.04	0.19
XBP1(S) activates chaperone genes	2	95	0.01	0.04	0.19
Chaperonin-mediated protein folding	2	96	0.01	0.04	0.19
Interleukin-12 family signaling	2	96	0.01	0.04	0.19

Diseases of programmed cell death	2	97	0.01	0.04	0.19
RAC3 GTPase cycle	2	100	0.01	0.05	0.19
IRE1alpha activates chaperones	2	101	0.01	0.05	0.19
Regulation of CDH11 mRNA translation by microRNAs	1	14	0.00	0.05	0.19
Protein folding	2	102	0.01	0.05	0.19

ESM Figures

ESM Figure 1

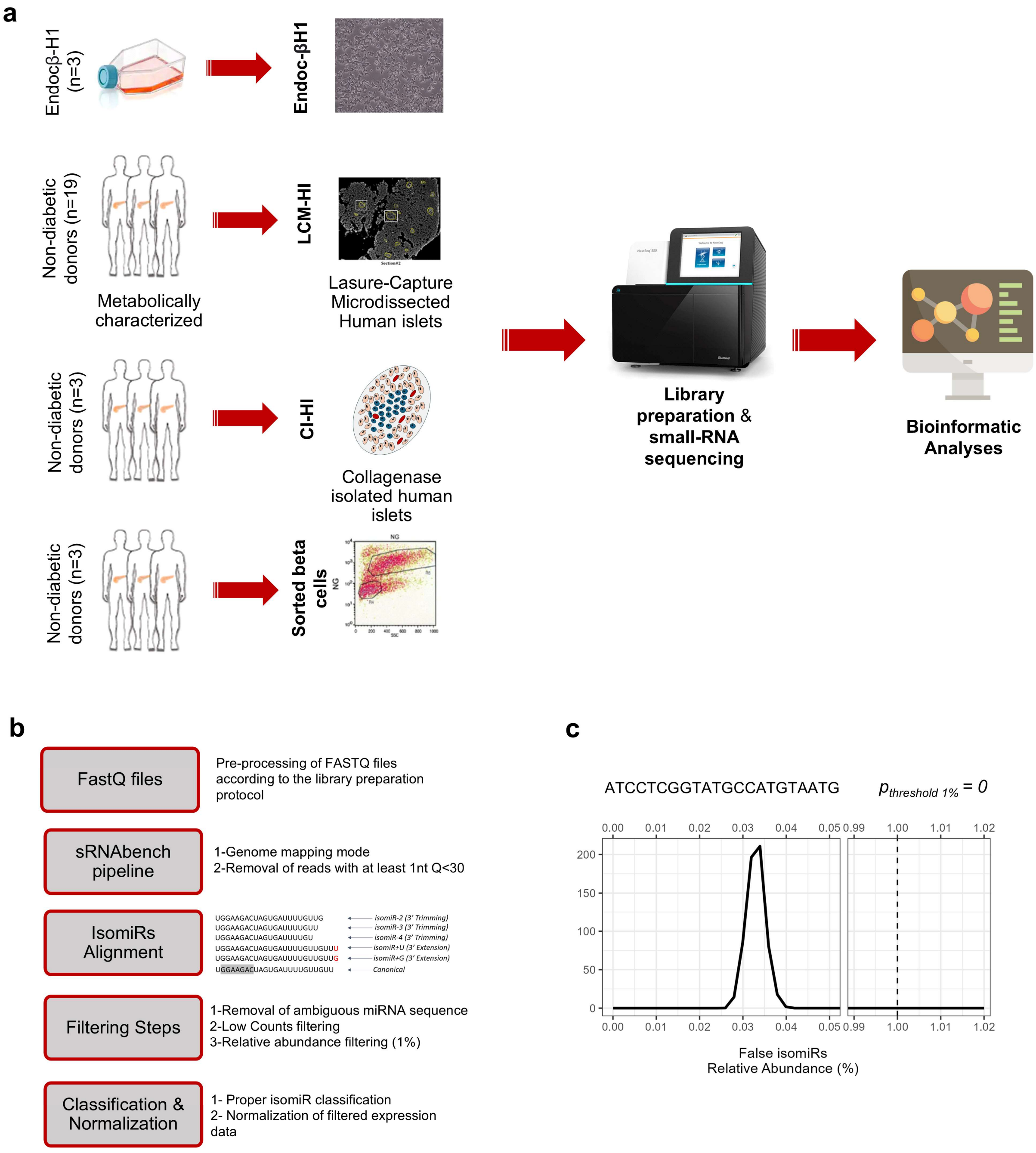

ESM Figure 1. Experimental Design and Bioinformatic analysis pipeline for the identification of isomiRs. (a) Study design for isomiR profiling in beta cells and human islets. RNA was extracted from (i) *n*=3 biological replicates of the EndoC-βH1 human beta cell line, (ii) laser capture microdissected human pancreatic islets (LCM-HI) from *n*=19 metabolically characterized non-diabetic donors, (iii) collagenase-isolated human islets (CI-HI) from *n*=3 non-diabetic donors, and (iv) fluorescence-activated cell sorting (FACS) sorted beta cells from *n*=3 non-diabetic donors. (**b**) Bioinformatics workflow for analyzing small RNA sequencing data. FastQ files were processed using the sRNAbench online pipeline in genome mapping mode, incorporating a sequence quality filter. Following alignment and quantification, additional filtering steps were applied to remove ambiguous sequences, low-expression sequences (low-count filtering), and sequences with minimal contribution to total miRNA expression (relative abundance filtering). Finally, the data were normalized, and sequences were classified into different isomiR classes based on post-transcriptional modifications. (**c**) Density plot from in-silico simulation showing the relative abundance distribution of false isomiR sequences with a single nucleotide variation compared to the reference sequence (*ATCCTCGGTATGCCATGTAATG*). The procedure was carried out simulating the reading the reference sequence 1.000.000 times, with a probability of error in the reading of each nucleotide = 0,1% (Q=30). The relative abundance threshold of 1% was validated by computing the probability of false isomiRs with a relative abundance above the cut-off (p=0).

ESM Figure 2

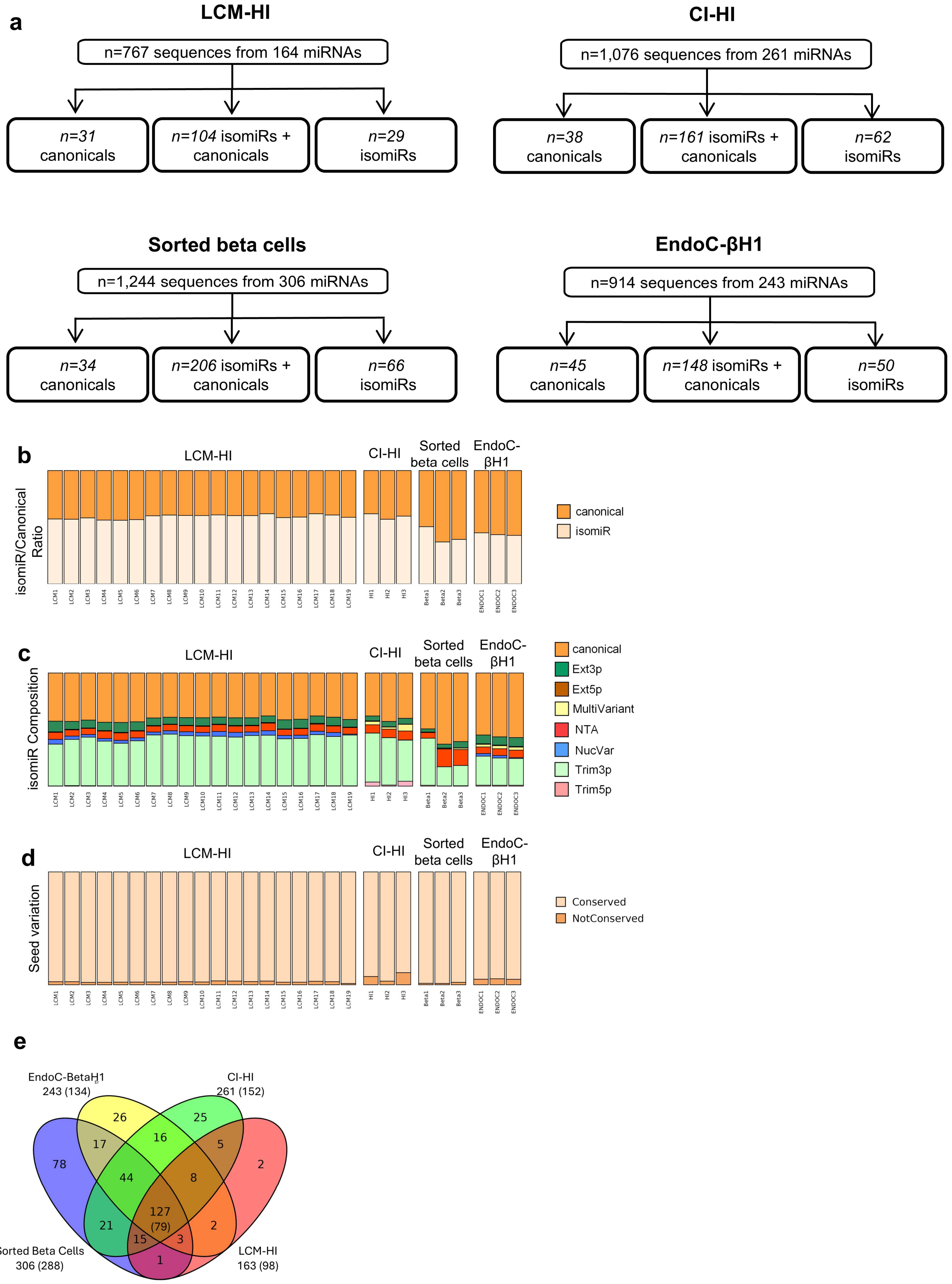
a
LCM-HI
n=767 sequences from 164 miRNAs
n=31 canonicals
n=104 isomiRs + canonicals
n=29 isomiRs
CI-HI
n=1,076 sequences from 261 miRNAs
n=38 canonicals
n=161 isomiRs + canonicals
n=62 isomiRs
Sorted beta cells
n=1,244 sequences from 306 miRNAs
n=34 canonicals
n=206 isomiRs + canonicals
n=66 isomiRs
EndoC-βH1
n=914 sequences from 243 miRNAs
n=45 canonicals
n=148 isomiRs + canonicals
n=50 isomiRs
b
LCM-HI
CI-HI
Sorted beta cells
EndoC-βH1
isomiR/Canonical Ratio
canonical
isomiR
c
isomiR Composition
canonical
Ext3p
Ext5p
MultiVariant
NTA
NucVar
Trim3p
Trim5p
d
Seed variation
Conserved
NotConserved
e
EndoC-BetaH1
243 (134)
CI-HI
261 (152)
Sorted Beta Cells
306 (288)
LCM-HI
163 (98)
26
25
17
16
5
78
44
8
2
127
(79)
21
2
15
3
1

ESM Figure 2. Canonical and isomiR sequences identification in human pancreatic islets and beta cells. (a) The scheme shows the distribution of the number of miRNA/isomiR sequences detected in CI-HI, LCM-HI, sorted beta cells, and EndoC-βH1 cells after the filtering steps. (**b-d**) Barplots showing the percentage of miRNAs/isomiRs counts (**b**), the percentage of different isomiRs classes (**c**), and the percentage of counts with and without conserved seed sequence (**d**) assigned to each sample in CI-HI, LCM-HI, sorted beta cells, and EndoC-βH1 cells. In LCM-human islet samples, LCM1 to LCM3 were obtained from non-diabetic multiorgan donors, while LCM4 to LCM19 were collected from non-diabetic living donors. (**e**) Venn diagram showing the intersection between the different miRNAs identified in the four experiments. In brackets, the number of miRNAs with total expression above 20 counts is represented. The final intersection of the four groups resulted in *n*=127 miRNAs identified in all the experiments, with *n*=79 having an expression above 20 counts.

ESM Figure 3

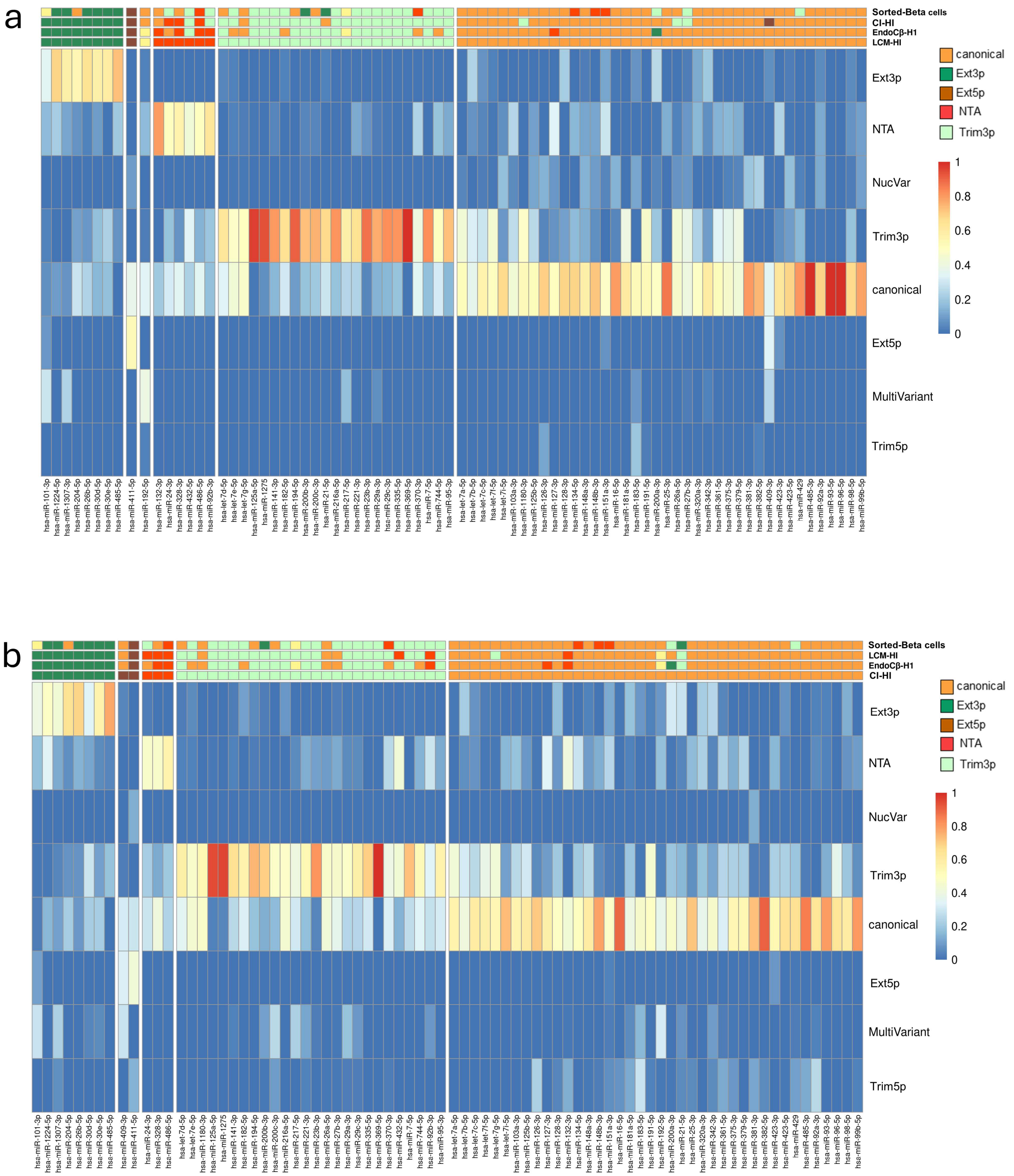

a
Sorted-Beta cells
CI-HI
EndoCβ-H1
LCM-HI
Ext3p
NTA
NucVar
Trim3p
canonical
Ext5p
MultiVariant
Trim5p
canonical
Ext3p
Ext5p
NTA
Trim3p
1
0.8
0.6
0.4
0.2
0
b
Sorted-Beta cells
LCM-HI
EndoCβ-H1
CI-HI

c

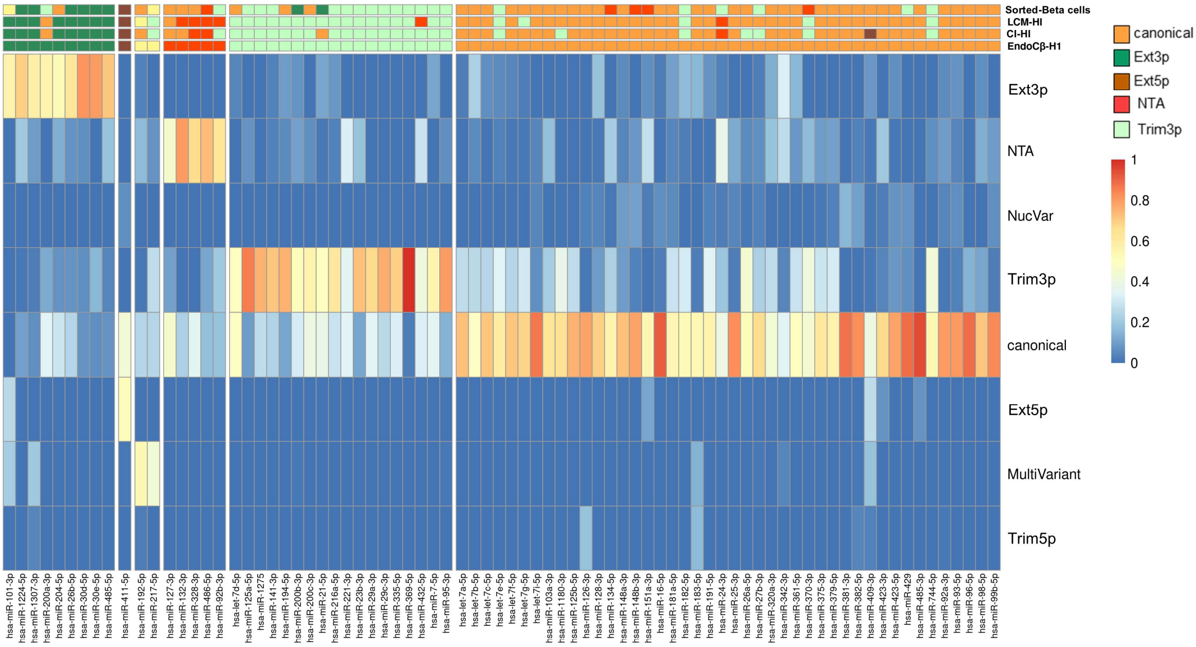

Sorted-Beta cells
LCM-HI
CI-HI
EndoCβ-H1
canonical
Ext3p
Ext5p
NTA
Trim3p
1
0.8
0.6
0.4
0.2
0
Ext3p
NTA
NucVar
Trim3p
canonical
Ext5p
MultiVariant
Trim5p

d

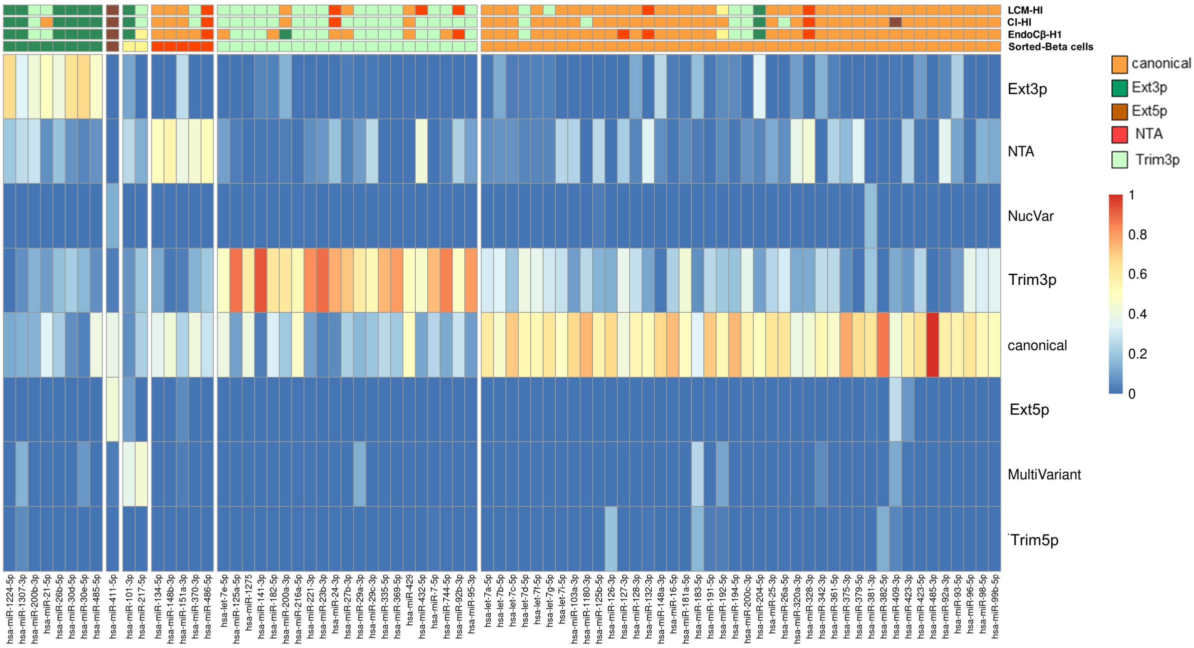

LCM-HI
CI-HI
EndoCβ-H1
Sorted-Beta cells
canonical
Ext3p
Ext5p
NTA
Trim3p
1
0.8
0.6
0.4
0.2
0
Ext3p
NTA
NucVar
Trim3p
canonical
Ext5p
MultiVariant
Trim5p

ESM Figure 3. Composition analysis in beta cell and human islets sequencing experiments. (a-d) Heatmap showing the proportion of counts (scaled 0 -1) assigned to the different isomiR classes in LCM-HI(**a**), CI-HI (**b**), EndoC-βH1 cells (**c**) and sorted beta cells (**d**). The top part of the heatmap reports the isomiR class accounting for most of the expression in the four beta cell and human islets sequencing experiments.

ESM Figure 4

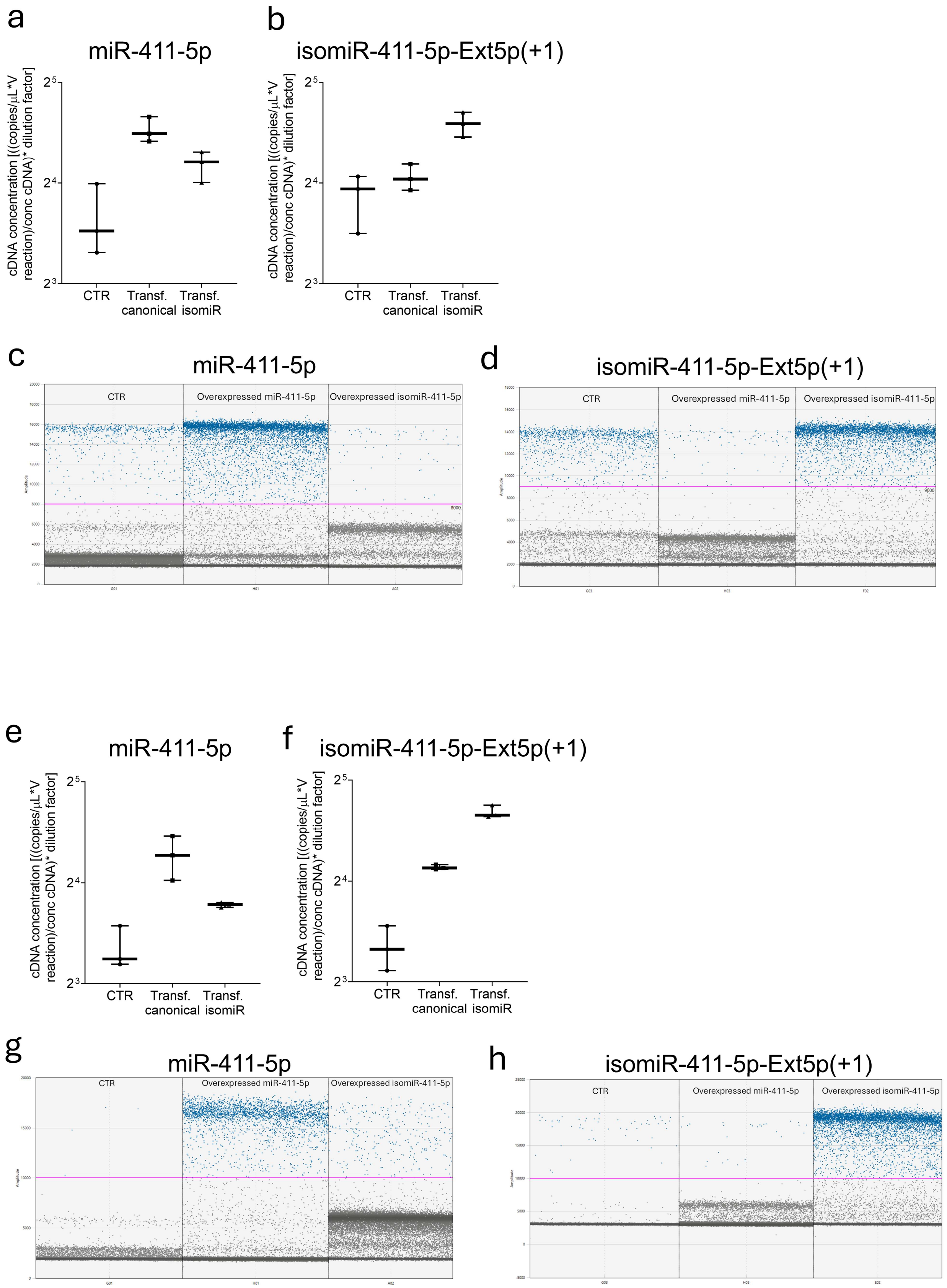
a
miR-411-5p
cDNA concentration [((copies/μL*V reaction)/conc cDNA)* dilution factor]
CTR
Transf. canonical
Transf. isomiR
b
isomiR-411-5p-Ext5p(+1)
c
miR-411-5p
CTR
Overexpressed miR-411-5p
Overexpressed isomiR-411-5p
d
isomiR-411-5p-Ext5p(+1)
e
miR-411-5p
f
isomiR-411-5p-Ext5p(+1)
g
miR-411-5p
h
isomiR-411-5p-Ext5p(+1)

ESM Figure 4. ddPCR Analysis of miR-411-5p and isomiR-411-5p-Ext5p(+1) upon their overexpression in EndoC-βH1 and CI-HI. (a-d) ddPCR analysis of miR-411-5p (a,c) and isomiR-411-5p-Ext5p(+1) (b,d) in EndoC-βH1 (*n*=3) transfected with miR-411-5p or isomiR-411-Ext5p(+1) mimics. Values are presented as log2 absolute copies +1. ddPCR droplet plots are reported in (c) and (d) showing the specific detection of isomiR but not miR-411 and viceversa. (e-h) ddPCR analysis of miR-411-5p (e,g) and isomiR-411-5p-Ext5p(+1) (f,h) in CI-HI (*n*=3) tranfected with miR-411-5p mimic or isomiR-411-5p-Ext5p(+1). ddPCR droplet plots are reported in (g) and (h) showing the specific detection of isomiR but not miR-411 and viceversa**.**

ESM Figure 5

a

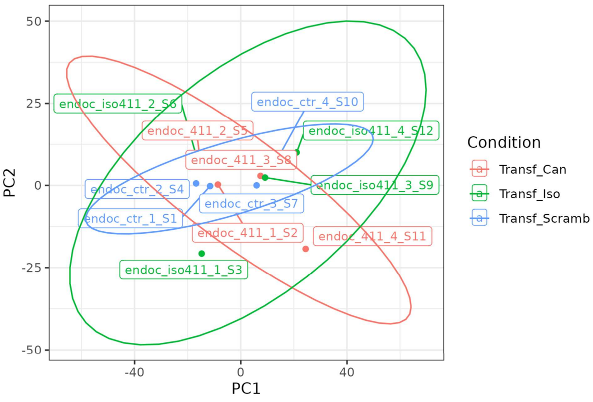

b

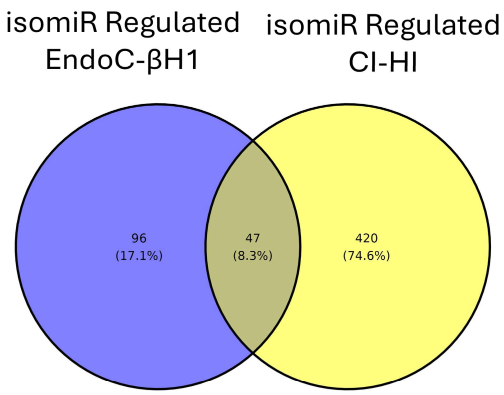

c

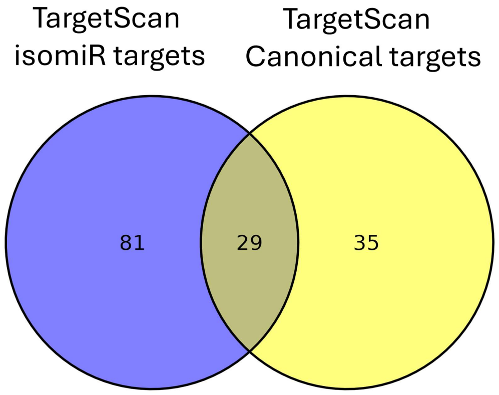

d

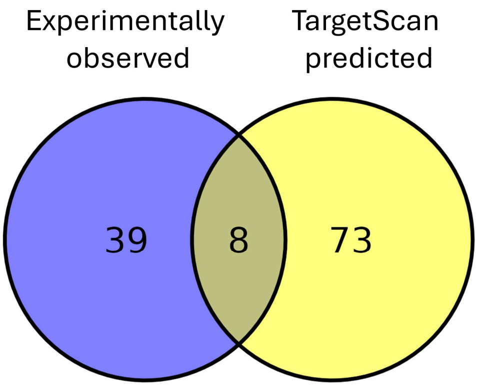

ESM Figure 5. isomiR-411-5p-Ext5p(+1) regulates a specific subset of transcripts. (**a**) Principal Components Analysis performed on RNA-sequencing data from the EndoC-βH1 cell line transfected with isomiR 411-5p-Ext5p(+1), canonical miR-411-5p and scrambled control. The ellipses showed no clear separation according to the different experimental conditions. The first 2 principal components are plotted. (**b**) Venn diagram showing the intersection between genes specifically downregulated by isomiR-411-5p-Ext5p(+1) both in EndoC-βH1 cell line and in CI-HI. (**c**) Venn diagram showing the intersection of the predicted targets genes of isomiR-411-5p-Ext5p(+1) and canonical-411-5p according to TargetScan. (**d**) Venn diagram showing the intersection between the signature of *n*=47 genes specifically downregulated by isomiR-411-5p-Ext5p(+1) both in EndoC-βH1 cell line and in CI-HI («Experimentally Observed») and the genes specifically targeted by isomiR-411-5p-Ext5p(+1) according to TargetScan prediction («TargetScan predicted»).

References

1. Brusco N, Sebastiani G, Di Giuseppe G, et al (2023) Intra-islet insulin synthesis defects are associated with endoplasmic reticulum stress and loss of beta cell identity in human diabetes. Diabetologia 66:354–366. https://doi.org/10.1007/s00125-022-05814-2

2. Mezza T, Sorice GP, Conte C, et al (2016) β-Cell Glucose Sensitivity Is Linked to Insulin/Glucagon Bihormonal Cells in Nondiabetic Humans. J Clin Endocrinol Metab 101:470–475. https://doi.org/10.1210/jc.2015-2802

3. American Diabetes Association (2022) Professional practice committee: *standards of medical care in diabetes—2022*. Diabetes Care 45:S3–S3. https://doi.org/10.2337/dc22-Sppc

4. Mezza T, Ferraro PM, Di Giuseppe G, et al (2021) Pancreaticoduodenectomy model demonstrates a fundamental role of dysfunctional β cells in predicting diabetes. J Clin Invest 131:. https://doi.org/10.1172/JCI146788

5. Mari A, Tura A, Natali A, et al (2010) Impaired beta cell glucose sensitivity rather than inadequate compensation for insulin resistance is the dominant defect in glucose intolerance. Diabetologia 53:749–756. https://doi.org/10.1007/s00125-009-1647-6

6. Ravassard P, Hazhouz Y, Pechberty S, et al (2011) A genetically engineered human pancreatic β cell line exhibiting glucose-inducible insulin secretion. J Clin Invest 121:3589–3597. https://doi.org/10.1172/JCI58447

7. Leinonen R, Sugawara H, Shumway M, International Nucleotide Sequence Database Collaboration (2011) The sequence read archive. Nucleic Acids Res 39:D19-21. https://doi.org/10.1093/nar/gkq1019

8. van de Bunt M, Gaulton KJ, Parts L, et al (2013) The miRNA profile of human pancreatic islets and beta-cells and relationship to type 2 diabetes pathogenesis. PLoS ONE 8:e55272. https://doi.org/10.1371/journal.pone.0055272

9. Parnaud G, Bosco D, Berney T, et al (2008) Proliferation of sorted human and rat beta cells. Diabetologia 51:91–100. https://doi.org/10.1007/s00125-007-0855-1

10. Grieco GE, Sebastiani G, Fignani D, et al (2021) Protocol to analyze circulating small non-coding RNAs by high-throughput RNA sequencing from human plasma samples. STAR Protocols 2:100606. https://doi.org/10.1016/j.xpro.2021.100606

11. Aparicio-Puerta E, Lebrón R, Rueda A, et al (2019) sRNAbench and sRNAtoolbox 2019: intuitive fast small RNA profiling and differential expression. Nucleic Acids Res 47:W530–W535. https://doi.org/10.1093/nar/gkz415

12. Frankish A, Diekhans M, Jungreis I, et al (2021) GENCODE 2021. Nucleic Acids Res 49:D916–D923. https://doi.org/10.1093/nar/gkaa1087

13. Kozomara A, Birgaoanu M, Griffiths-Jones S (2019) miRBase: from microRNA sequences to function. Nucleic Acids Res 47:D155–D162. https://doi.org/10.1093/nar/gky1141

14. Robinson MD, McCarthy DJ, Smyth GK (2010) edgeR: a Bioconductor package for differential expression analysis of digital gene expression data. Bioinformatics 26:139–140. https://doi.org/10.1093/bioinformatics/btp616

15. Love MI, Huber W, Anders S (2014) Moderated estimation of fold change and dispersion for RNA-seq data with DESeq2. Genome Biol 15:550. https://doi.org/10.1186/s13059-014-0550-8

16. Martin M (2011) Cutadapt removes adapter sequences from high-throughput sequencing reads. EMBnet j 17:10. https://doi.org/10.14806/ej.17.1.200

17. Aparicio-Puerta E, Hirsch P, Schmartz GP, et al (2023) isomiRdb: microRNA expression at isoform resolution. Nucleic Acids Res 51:D179–D185. https://doi.org/10.1093/nar/gkac884

18. Ewels P, Magnusson M, Lundin S, Käller M (2016) MultiQC: summarize analysis results for multiple tools and samples in a single report. Bioinformatics 32:3047–3048. https://doi.org/10.1093/bioinformatics/btw354

19. Chen S, Zhou Y, Chen Y, Gu J (2018) fastp: an ultra-fast all-in-one FASTQ preprocessor. Bioinformatics 34:i884–i890. https://doi.org/10.1093/bioinformatics/bty560

20. Dobin A, Davis CA, Schlesinger F, et al (2013) STAR: ultrafast universal RNA-seq aligner. Bioinformatics 29:15–21. https://doi.org/10.1093/bioinformatics/bts635

21. Smith T, Heger A, Sudbery I (2017) UMI-tools: modeling sequencing errors in Unique Molecular Identifiers to improve quantification accuracy. Genome Res 27:491–499. https://doi.org/10.1101/gr.209601.116

22. Liao Y, Smyth GK, Shi W (2014) featureCounts: an efficient general purpose program for assigning sequence reads to genomic features. Bioinformatics 30:923–930. https://doi.org/10.1093/bioinformatics/btt656

23. Fabregat A, Korninger F, Viteri G, et al (2018) Reactome graph database: Efficient access to complex pathway data. PLoS Comput Biol 14:e1005968. https://doi.org/10.1371/journal.pcbi.1005968

24. Lewis BP, Burge CB, Bartel DP (2005) Conserved seed pairing, often flanked by adenosines, indicates that thousands of human genes are microRNA targets. Cell 120:15–20. https://doi.org/10.1016/j.cell.2004.12.035

25. Cinelli C, Ferwerda J, Hazlett C (2020) Sensemakr: sensitivity analysis tools for OLS in R and stata. SSRN Journal. https://doi.org/10.2139/ssrn.3588978

Diabetologia

Checklist for reporting human islet preparations used in research

Adapted from Hart NJ, Powers AC (2018) Progress, challenges, and suggestions for using human islets to understand islet biology and human diabetes. Diabetologia https://doi.org/10.1007/s00125-018-4772-2

**Islet preparation**	**1**	**2**	**3**	**4**	**5**	**6**	**7**	**8[a]**
**MANDATORY INFORMATION**								
Unique identifier	HP-24024-01	HP-24067-01	HP-24151-01	HI9	HI12	HI7		
Donor age (years)	56	55	38	59	47	54		
Donor sex (M/F)	F	M	M	F	F	M		
Donor BMI (kg/m^2)	32,4	29,9	23,2	23,7	33,4	28,5		
Donor HbA_{1c} or other measure of blood glucose control	5,8	5,6	5,7	n/a	n/a	n/a		
Origin/source of islets[b]	TebuBio	TebuBio	TebuBio	ECIT	ECIT	ECIT		
Islet isolation centre	Prodo Labs	Prodo Labs	Prodo Labs	University of Geneva	University of Geneva	University of Geneva		
Donor history of diabetes? Please select yes/no from drop down list	No	No	No	No	No	No		
**If Yes, complete the next two lines if this information is available**								
Diabetes duration (years)								
Glucose-lowering therapy at time of death[c]								
**RECOMMENDED INFORMATION**								
Donor cause of death	Stroke	Anoxic Event	Anoxic Event	Cerebral trauma	Cerebral haemorrage	Cerebral trauma		
Warm ischaemia time (h)								
Cold ischaemia time (h)								
Estimated purity (%)	90-95	90	90	84	88	60		
Estimated viability (%)	95	95	95	90	90	89		
Total culture time (h)[d]								
Glucose-stimulated insulin secretion or other functional measurement[e]								
Handpicked to purity? Please select yes/no from drop down list								
Additional notes								

[a]If you have used more than eight islet preparations, please complete additional forms as necessary
[b]For example, IIDP, ECIT, Alberta IsletCore
[c]Please specify the therapy/therapies
[d]Time of islet culture at the isolation centre, during shipment and at the receiving laboratory
[e]Please specify the test and the results
